# Orally Active Peptide Vector Allows Using Cannabis to Fight Pain While Avoiding Side Effects

**DOI:** 10.1021/acs.jmedchem.1c00484

**Published:** 2021-04-23

**Authors:** Maria Gallo, Estefanía Moreno, Sira Defaus, Antonio Ortega-Alvaro, Angel Gonzalez, Patricia Robledo, Marco Cavaco, Vera Neves, Miguel A. R. B. Castanho, Vicent Casadó, Leonardo Pardo, Rafael Maldonado, David Andreu

**Affiliations:** † Department of Experimental and Health Sciences, Universitat Pompeu Fabra, Barcelona Biomedical Research Park, 08003 Barcelona, Spain; ‡ Department of Biochemistry and Molecular Biomedicine, Institute of Biomedicine, University of Barcelona, 08028 Barcelona, Spain; § Laboratori de Medicina Computacional, Unitat de Bioestadística, Facultat de Medicina, Universitat Autònoma de Barcelona, 08193 Bellaterra, Spain; ∥ Integrative Pharmacology and Systems Neuroscience, IMIM-Hospital del Mar Research Institute, 08003 Barcelona, Spain; ⊥ Instituto de Medicina Molecular, Faculdade de Medicina, Universidade de Lisboa, 1649-028 Lisboa, Portugal

## Abstract

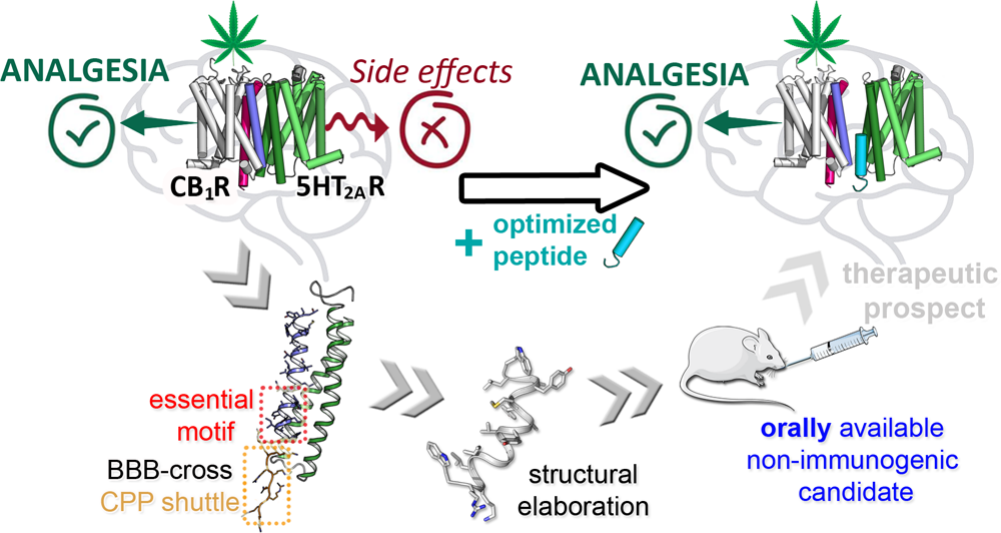

The activation of cannabinoid CB_1_ receptors (CB_1_R) by Δ^9^-tetrahydrocannabinol (THC), the main component of *Cannabis sativa*, induces analgesia. CB_1_R activation, however, also causes cognitive impairment *via* the serotonin 5HT_2A_ receptor (5HT_2A_R), a component of a CB_1_R–5HT_2A_R heteromer, posing a serious drawback for cannabinoid therapeutic use. We have shown that peptides reproducing CB_1_R transmembrane (TM) helices 5 and 6, fused to a cell-penetrating sequence (CPP), can alter the structure of the CB_1_R–5HT_2A_R heteromer and avert THC cognitive impairment while preserving analgesia. Here, we report the optimization of these prototypes into drug-like leads by (i) shortening the TM5, TM6, and CPP sequences, without losing the ability to disturb the CB_1_R–5HT_2A_R heteromer, and (ii) extensive sequence remodeling to achieve protease resistance and blood–brain barrier penetration. Our efforts have culminated in the identification of an ideal candidate for cannabis-based pain management, an orally active 16-residue peptide preserving THC-induced analgesia.

## Introduction

About 20% of the population suffers from chronic pain that seriously affects personal and professional life, highlighting the urgent need for analgesics that effectively alleviate pain with minimal side effects.^1^ Opioids, thus far the mainstay in severe pain management, are under intense debate for their poor safety profiles and high potential for abuse, as reflected in the opioid abuse disorder epidemic claiming about 145 deaths/day in the recent years in the USA.^2,3^ On the other hand, patients with minor pain manifestations are usually dealt with nonsteroidal anti-inflammatories (NSAIDs) that not only fail quite often to achieve adequate relief but also have serious side effects (*e.g.*, gastrointestinal and renal).^4^ Thus, there is a need to fill the therapeutic gap between opioids and NSAIDs under multiple moderate pain conditions or in situations where opioids are ineffective, such as severe neuropathic pain.^5^

The analgesic potential of cannabinoid compounds has long been recognized.^6−8^ The activation of cannabinoid CB_1_ receptors (CB_1_R) by agonists, such as Δ^9^-tetrahydrocannabinol (THC), the main psychoactive component of *Cannabis sativa*, elicits therapeutically relevant responses for the treatment of migraine,^9,10^ rheumatoid arthritis,^10^ osteoarthritis,^11^ neuropathic,^12^ or cancer-related pain,^13^ among many others. Unfortunately, CB_1_R activation is also linked to side effects, such as memory impairment, with major consequences in cannabis users.^8,14−17^ These adverse effects pose a serious hurdle to the therapeutic use of cannabinoids; hence, the possibility to minimize their impact would constitute a major breakthrough.

Behavioral studies in mice lacking the serotonin 5HT_2A_ receptor (5HT_2A_R) revealed a remarkable dissociation between the beneficial antinociceptive effects of THC and its detrimental amnesic properties.^18^ As CB_1_R and 5HT_2A_R are G protein-coupled receptors (GPCRs), a class of proteins frequently forming oligomers in cells,^19^ we provided evidence that the underlying molecular basis for that observation was the formation of CB_1_R–5HT_2A_R heteromers.^18^ Both receptors are functionally active in specific brain regions; the heteromer shows specific biochemical signatures distinct from those of the protomers, in artificial cell lines as well as native tissues; very importantly, while the cognitive impairment caused by THC is due to the CB_1_R–5HT_2A_R heteromer, the antinociceptive effects result solely from CB_1_R activation.^18^ The arrangement of CB_1_R and 5HT_2A_R protomers in the heteromer is *via* transmembrane (TM) helices 5 and 6^18^ as studied by bimolecular fluorescence complementation (BiFC) experiments with synthetic peptides **TM5-Tat** and **Tat-TM6**, where TM5 and 6 helices are fused to cell-penetrating peptide (CPP) GRKKRRQRRR, that is, HIV Tat(48–57),^20^ respectively, at N- or C-termini, ensuring TM native alignment relative to the membrane.

The formation of a stable four-helix bundle between these TM5 and 6 helices of both protomers, as described for the dimeric crystal of the μ-opioid receptor,^21^ was suggested to be involved in the allosteric interactions between protomers and in the mechanisms behind the specific signaling signatures of the heteromer.^18,22^ Moreover, interfering **TM5-Tat** and **Tat-TM6** peptides were able to alter the structure of the heteromer *in vivo*, leading to a selective abrogation of memory impairments caused by the exposure to THC.^18^ From this, it was logical to assume that disturbing the formation of the CB_1_R–5HT_2A_R heteromer might be an effective strategy for harnessing the therapeutic potential of THC while avoiding its side effects. **TM5-Tat** and **Tat-TM6** peptides, however, were ungainly therapeutic leads with poor prospects related to their large size, low solubility, and inability to cross the blood–brain barrier (BBB). We now report on a comprehensive optimization effort leading from those prototypes to a candidate with vastly improved, drug-like features (Table 1) including small size, proteolytic stability, BBB crossing ability, and, remarkably, oral activity, thereby achieving the desired effects, both *in vitro* and *in vivo*.

**Table 1:** From TM5/6 Prototypes to a Drug-Like Finalist Peptide^a^

peptide		sequence^b^	residues
prototypes^18^	**TM5-Tat**	ETYLMFWIGVTSVLLLFIVYAYMYILWGRKKRRQRRR	37
	**Tat-TM6**	GRKKRRQRRRKTLVLILVVLIICWGPLLAIMVYDVF	36
optimized candidate		wliymyayvaGilkrw	16

a A detailed list of all peptides used in the various rounds of the optimization process is given in Tables S1–S4.

b Upper and lower case denote l- and d-residues, respectively.

## Results

### Shorter Peptides Mimicking CB_1_R TM Helices **5** and **6** and Altering CB_1_R–5HT_2A_R Heteromers

**Figure 1:**
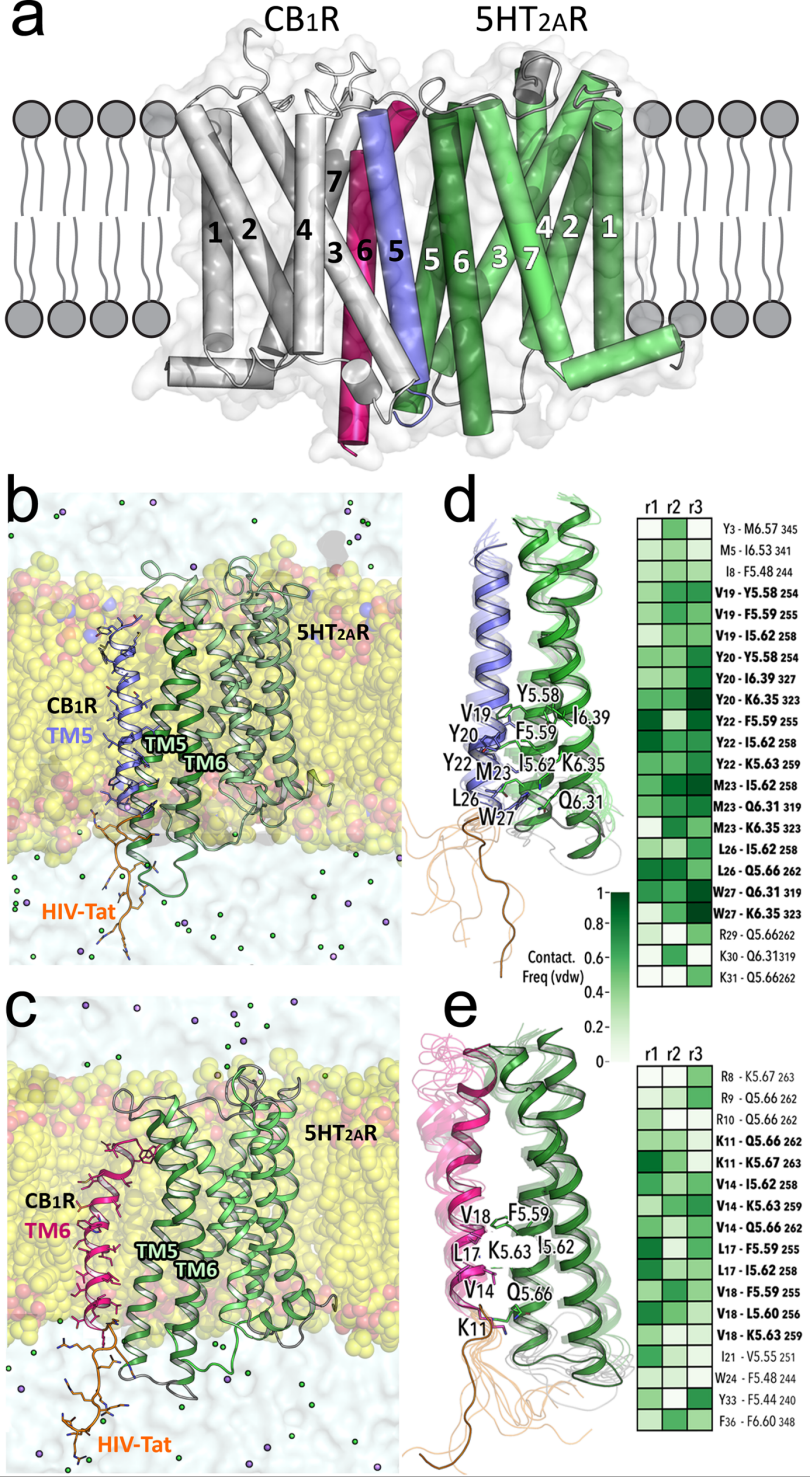
(a–e) MD simulations of 5HT_2A_R in complex with **TM5-Tat** and **Tat-TM6** peptides. The previously published computational model of the CB_1_R–5HT_2A_R heteromer^18,20^ (panel a) was used as the starting point for MD simulations (see Experimental Section) of a membrane-embedded (yellow spheres) 5HT_2A_R (green ribbons) in complex with peptides where the TM5 (blue ribbon, panel b) or TM6 (red ribbon, panel c) sequences are fused to HIV-Tat(48–57) (orange). These **TM5-Tat** and **Tat-TM6** peptides (Table 1) were stable in the three replicas of 1 μs unbiased MD, as shown by root-mean-square deviations (Figure S1). Panels (d,e) show detailed views of the interactions between **TM5-Tat** and **Tat-TM6**, respectively, with TM helices 5 and 6 of 5HT_2A_R. Snapshots along the trajectories are shown as transparent ribbons, whereas representative structures are solid ribbons. Side chain residues involved in stable interactions of **TM5-Tat** and **Tat-TM6** with 5HT_2A_R (Ballesteros–Weinstein notation) during the MD simulations are labeled. Interaction frequency maps for frequencies above 50% in at least one of the three replicas (r1, r2, and r3) are depicted. **TM5-Tat** and **Tat-TM6** residues involved in stable interactions with two or more 5HT_2A_R residues are highlighted in bold.

The design of these peptides required the identification of the amino acid residues in **TM5-Tat** and **Tat-TM6** that contribute more significantly to the binding to 5HT_2A_R. This was studied by several replicas of unbiased 1 μs molecular dynamic (MD) simulations of **TM5-Tat** and **Tat-TM6** in complex with 5HT_2A_R (see Figure 1a–c and Experimental Section). The simulations show that the intracellular part of the peptides remains more stable than the extracellular one (more noticeable for **Tat-TM6**) due to specific interactions with 5HT_2A_R (mainly the V19-Y20-Y22-M23-L26-W27 stretch in **TM5-Tat** or the K11-V14-L17-V18 stretch in **Tat-TM6**, Figure 1d,e).

**Figure 2:**
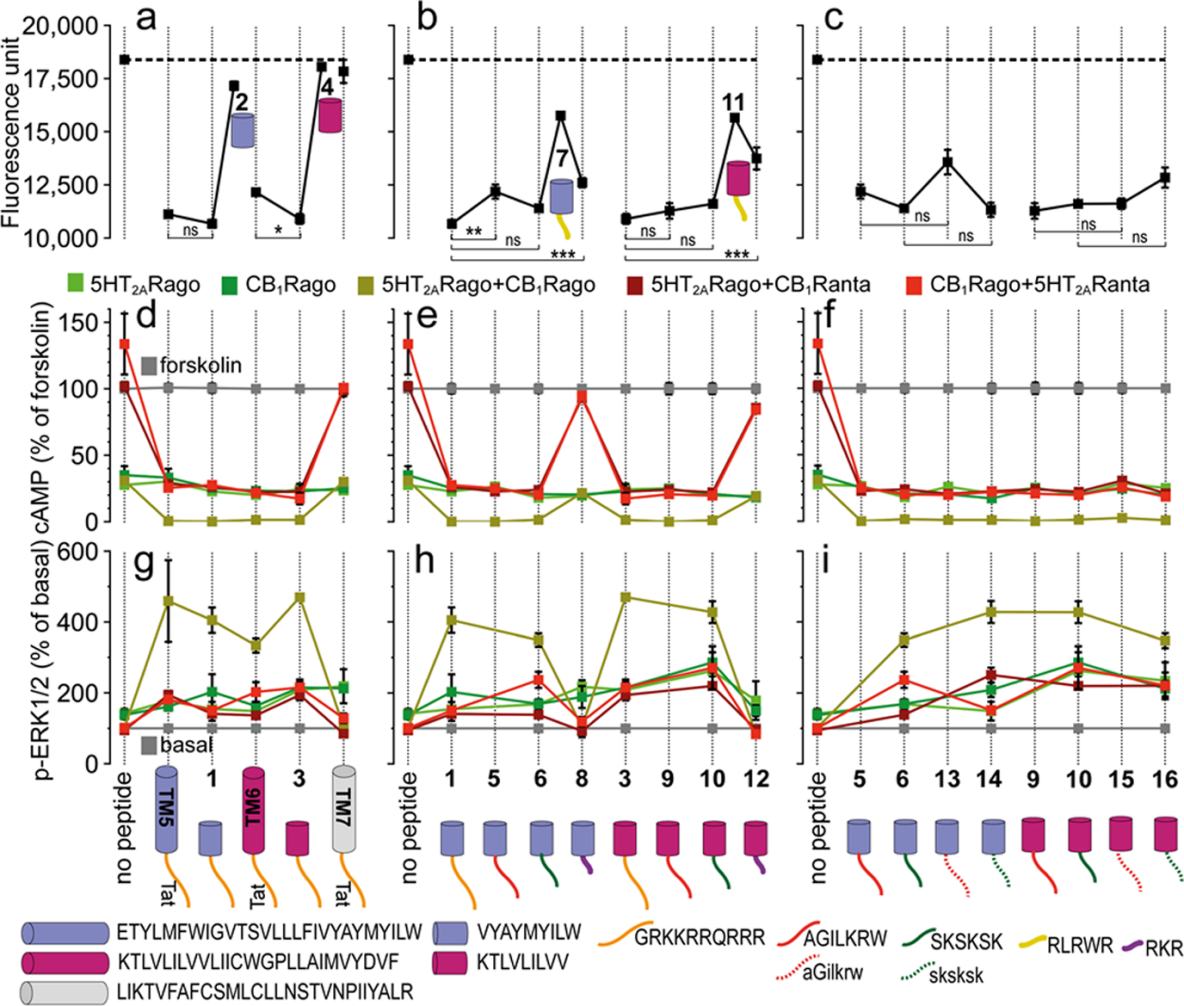
(a–c) BiFC analysis of the effect of designed peptides on CB_1_R–5HT_2A_R heteromerization. Fluorescence (530 nm) of HEK-293T cells transfected with 5HT_2A_R-cYFP and CB_1_R-nYFP treated with a vehicle (no peptide) or 4 μM peptide for 4 h. Values are mean ± SEM of *n* = 10–30; **TM5-Tat** and **Tat-TM6** are positive controls, whereas **TM7-Tat** is a negative control; ns (no significant), *(*p* < 0.05), **(*p* < 0.01), and ***(*p* < 0.001) represent significantly different (two-tailed) values (one-way ANOVA followed by Tukey’s multiple comparison tests). (d–i) Effect of designed peptides on the decrease in FK-induced cAMP (d–f) or increase in ERK1/2 phosphorylation. (g–i) Transfected cells preincubated with a vehicle (no peptide) or with 5HT_2A_R (5HT_2A_Rago, DOI, 100 nM) or CB_1_R (CB_1_Rago, WIN, 100 nM) agonists or with 5HT_2A_R (5HT_2A_Ranta, MDL, 300 nM) or CB_1_R (CB_1_Ranta, RIM, 1 μM) antagonists and combinations thereof, in the presence or absence of 0.5 μM FK. Values in panels (d–f) are mean ± SEM of *n* = 3–6 of FK-treated cells. Quantification of phosphorylated ERK-1/2 was determined by the α-screen bead-based technology. Values in panels (g–i), expressed as a percentage of basal (nontreated cells), are mean ± SEM of *n* = 4–9. Two-way ANOVA followed by Tukey’s multiple comparison tests was used to analyze the data (Table S5). Cartoons depict the designed peptides (cylinders with color codes as in Figure 1) fused to different CPPs (lines).

Based on these predictions,^18^ peptides VYAYMYILW-Tat and Tat-KTLVLILVV (**1** and **3**, Table S1), respectively, mimicking the key interactions of **TM5-Tat** and **Tat-TM6**, were designed. Their ability to alter CB_1_R–5HT_2A_R heteromer formation was first tested by BiFC assays,^18,23,24^ in HEK-293T cells transfected with receptors fused to two complementary halves of YFP (5HT_2A_R-cYFP and CB_1_R-nYFP). In this assay, **1** and **3** caused a decrease in fluorescence complementation comparable to the **TM5-Tat** and **Tat-TM6** prototypes, in contrast to peptide **TM7-Tat**, used as a negative control (Figure 2a). Moreover, the biochemical properties of the heteromer differ from those of the protomers.^18,23,24^ In CB_1_R–5HT_2A_R, in addition to a G_q_ to G_i_ switch in 5HT_2A_R coupling preference, antagonist binding to one of the receptors blocks signaling of the interacting receptor (known as cross-antagonism), and costimulation with both agonists does not produce an additive effect (known as negative cross-talk).^23,24^ Thus, we next determined signaling after receptor activation by measuring cAMP levels [decrease in forskolin (FK)-induced cAMP as the result of adenylate cyclase inhibition by G_i_] or the increase in pERK in the ERK1/2 phosphorylation pathway (Figure 2d,g). Cells stimulated with FK and treated with the CB_1_R agonist WIN 55,212-2 (WIN) or 5HT_2A_R agonist 2,5-dimethoxy-4-iodoamphetamine (DOI) showed reduced cAMP production in all groups, as expected for G_i_-coupled receptors. Neither **TM5-Tat**, **Tat-TM6**, peptides **1** or **3**, nor **TM7-Tat** (negative control) influenced G protein coupling preferences (5HT_2A_R remains G_i_-coupled in the presence of interfering peptides). The CB_1_R antagonist rimonabant (RIM) blocked the decrease in FK-induced cAMP or the increase in pERK triggered by the 5HT_2A_R agonist DOI, and the 5HT_2A_R antagonist MDL 100,907 (MDL) also blocked the decrease in cAMP and increase in pERK induced by the CB_1_R agonist WIN (bidirectional cross-antagonism). Notably, peptides **1** and **3** eliminated this bidirectional cross-antagonism as efficiently as **TM5-Tat** and **Tat-TM6** peptides, an effect that was not observed with **TM7-Tat** (negative control). Coadministration of WIN and DOI agonists, in the absence of peptides, does not lead to a further statistically significant decrease in cAMP or increase in pERK (negative cross-talk). However, in the presence of **1** and **3**, as well as **TM5-Tat** and **Tat-TM6** (but not **TM7-Tat**), the simultaneous addition of both agonists results in a significant decrease in cAMP or an increase in pERK (absence of negative cross-talk). In sum, interfering peptides **1** and **3** successfully blocked key biochemical signatures (cross-antagonism and negative cross-talk) of the CB_1_R–5HT_2A_R heteromer as efficiently as **TM5-Tat** and **Tat-TM6**.

We assumed that the segments of peptides **TM5-Tat** and **Tat-TM6** replicating CB_1_R TM helices 5 and 6 are membrane-embedded as in the receptor, whereas Tat(48–57) is in the intracellular space (Figure 1b,c). Peptides **2** and **4** (Table S1), lacking the Tat(48–57) sequence, had no effect on decreasing fluorescence complementation (Figure 2a) or in signaling (Figure 2d–g), underscoring the essential role of the CPP motif.

### HIV-Tat Replacement by Shorter CPPs

Inspired by recent studies,^25−28^ a second set of analogues were devised (Table S2) in which four alternatives to 10-residue Tat(48–57) were explored: (i) 7-residue PepH3 (AGILKRW), a reported BBB shuttle^25^ (peptides **5** and **9**); (ii) 6-residue SKSKSK^26^ (**6** and **10**); (iii) 5-residue RLRWR^27^ (**7** and **11**), and (iv) 3-residue RKR^28^ (**8** and **12**). The last two CPP motifs (**7**, **8**, **11**, and **12**) were not as efficient as Tat(48–57) in decreasing fluorescence complementation, as measured in BiFC assays (Figure 2b), neither could **8** or **12** abolish the cross-antagonism and negative cross-talk signatures of the CB_1_R–5HT_2A_R heteromer (Figure 2e,h). In contrast, peptides **5**/**9** and **6**/**10** were as effective as peptides **1/3** in fluorescence complementation or signaling (Figure 2b,e,h) and were thus selected for further optimization.

### CPP Stereochemistry and Its Significance

Replacement of natural l-by d-amino acids results in CPP sequences with improved cell entry and metabolic stability.^29,30^ We thus switched the CPP motifs of peptides **5**, **6**, **9**, and **10** to their enantiomeric d-versions, while preserving the L configuration for the cell membrane-embedded sections replicating CB_1_R TM5 or 6 (peptides **13**–**16**, Table S3). Trypsin incubation over 24 h confirmed our expectations of improved proteolytic stability in the **13**–**16** set, in contrast to poor survival for all-l counterparts (**5**, **6**, **9**, and **10**) (Figure S2a). More relevantly, **13–16** maintained the CB_1_R–5HT_2A_R heteromer-disturbing ability (Figure 2c) and blocking of cross-antagonism and negative cross-talk (Figure 2f,i), as somehow expected from the stereochemical nonmodification of the membrane-embedded TM segments.

### BBB Translocation

In our earlier report,^18^ tacitly assuming the inability of **TM5-Tat** and **Tat-TM6** to traverse the BBB, these peptides were administered by intracerebroventricular (ICV) infusion, an invasive route of little interest for human therapeutic application. However, for a peptide drug to be a successful candidate, BBB-crossing capability is mandatory. Therefore, to explore the BBB-crossing features of optimized peptides **13–16**, their 5(6)-carboxyfluorescein-labeled versions (see Experimental Section) were tested for translocation in an *in vitro* BBB model using mouse b.End3 cells.^30,31^ Fluorescence intensity readings at the basolateral chamber of the transwell device showed that **13** and **15**, with the TM-altering sequence fused to d-CPP motif aGilkrw, translocated more efficiently than **14** and **16**, where the TM-altering sequence was fused to the sksksk vector (Figure S2b). The high *in vitro* BBB integrity observed after 24 h incubation ruled out paracellular transport (Figure S2c). In parallel, cytotoxicity studies on b.End3 cells were conducted to discard potential toxic effects modifying barrier permeability, that is, artifactual peptide ability to cross the BBB (Figure S2d).

### Applying Configurational Switch and Sequence Reversal for Last-Stage Lead Optimization

**Figure 3:**
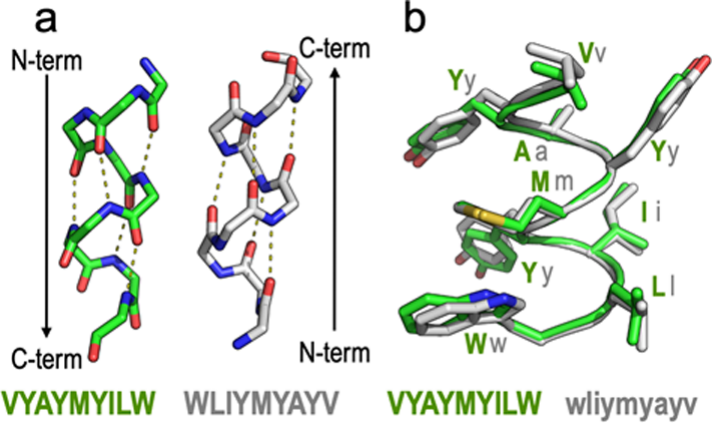
(a) Retro modification of a short TM5 peptide (VYAYMYILW) where the peptide sequence has been synthesized in the reverse order (WLIYMYAYV). (b) When the enantio modification (d-amino acids) is additionally applied (wliymyayv), the orientation of the side chains is similar to that of the original peptide.

Although considerable progress had been achieved in terms of BBB-crossing, in the CPP-enabled constructs delivering short TM payloads to the CB_1_R–5HT_2A_R heteromer, the essential modifying segments (*i.e.*, short TM5: VYAYMYILW and short TM6: KTLVLILVV) were still made up of natural l-amino acids, susceptible to endopeptidases such as chymotrypsin, thermolysin, or pepsin, targeting preferentially hydrophobic and/or aromatic residues (Ile, Leu, Val, Ala, Met, Trp, and Tyr) whose abundance in those TM sequences was an important concern. To boost protease resistance, both in body fluids and within the target cell, we developed two last-stage candidates (peptides **17–18**, Table S4) where the optimal BBB-permeable shuttle (*i.e.*, aGilkrw) was fused to the retro-enantio (r/e) versions of the TM5- and TM6-altering sequences (*i.e.*, short TM5 and short TM6 composed of d-amino acids in the reversed sequence). In such an arrangement (Figure 3), if the all-l sequence is displayed in the conventional N- to C-terminal fashion but the r/e analogue is laid out in the opposite (C- to N-terminal) sense by way of a 180° rotation in the plane, the side chains adopt similar orientations,^32−35^ although the amide bonds show inverted (CONH *vs* NHCO) directions. In summary, peptides **17–18**, recapitulating the various rounds of structural elaboration so far, met a priori all desired requirements, including (i) the same TM5/TM6 side chain orientation than previous CB_1_R–5HT_2A_R-modifying analogues, (ii) high proteolytic resistance as expected from an all-d composition,^36^ and (iii) ability to traverse the BBB.^30^

**Figure 4:**
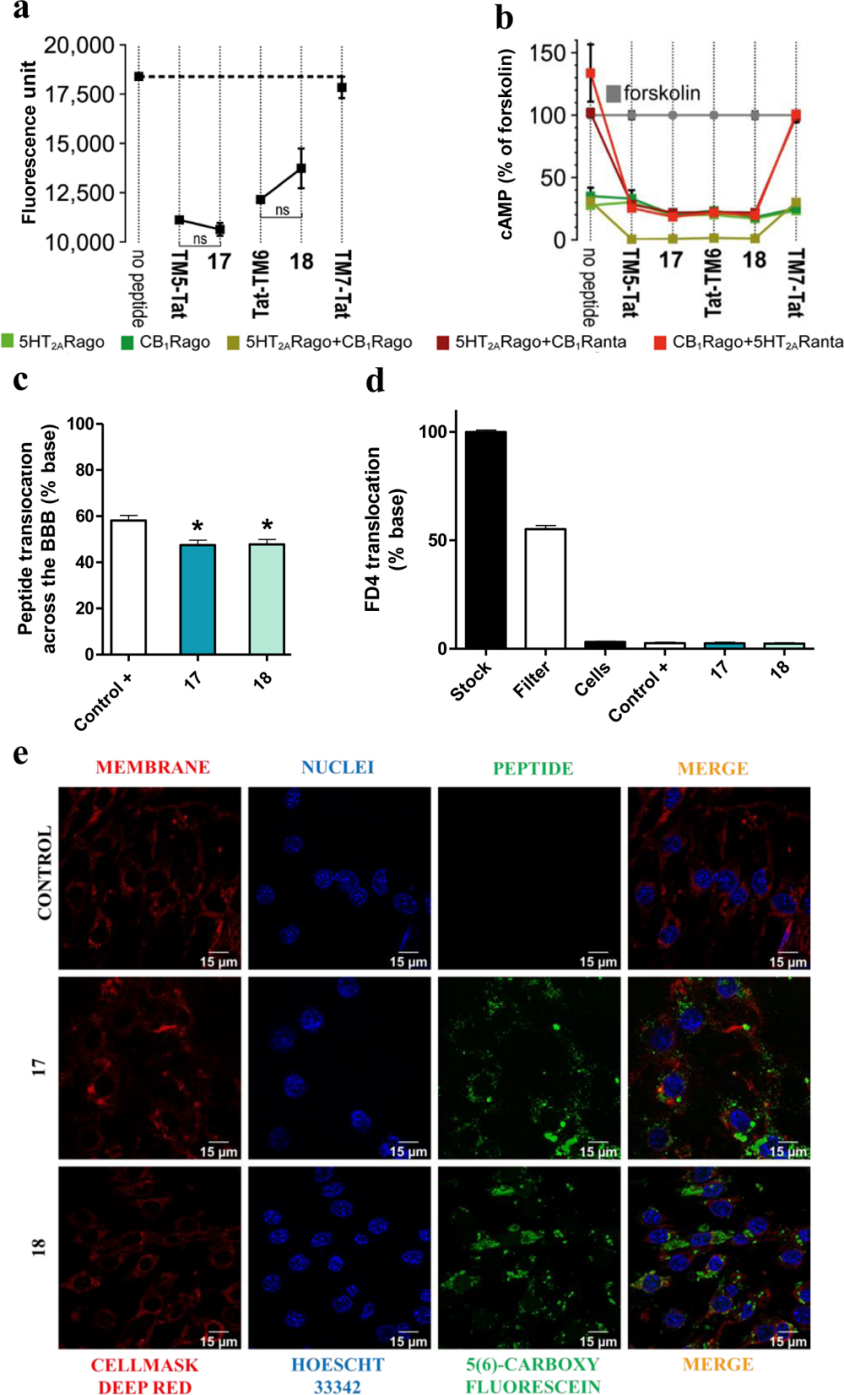
(a) BiFC analysis of the effect of peptides **17** and **18** on CB_1_R–5HT_2A_R heteromerization. Fluorescence (530 nm) of HEK-293T cells transfected with 5HT_2A_R-cYFP and CB_1_R-nYFP treated with a vehicle or peptide. Values are mean ± SEM of *n* = 10–30. **TM5-Tat** and **Tat-TM6** are positive controls, whereas **TM7-Tat** is a negative control; ns (no significant), (one-way ANOVA followed by Tukey’s multiple comparison tests). (b) Effect of peptides **17** and **18** on cAMP production. Transfected cells preincubated with a vehicle (no peptide) or with 5HT_2A_R (5HT_2A_Rago, DOI, 100 nM) or CB_1_R (CB_1_Rago, WIN, 100 nM) agonists or with 5HT_2A_R (5HT_2A_Ranta, MDL, 300 nM) or CB_1_R (CB_1_Ranta, RIM, 1 μM) antagonists and combinations thereof, in the presence of FK. Values are mean ± SEM of *n* = 3–6 of FK-treated cells. Two-way ANOVA followed by Tukey’s multiple comparison tests was used to analyze the data (Table S5). (c) *In vitro* translocation of peptides **17** and **18**. Translocation (%) across the transwell BBB model quantified as fluorescence in the basolateral chamber after 24 h. A trans-BBB peptide was used as the positive control. Values are mean ± SEM of *n* = 3–6. *p < 0.05 *vs* positive control (one-way ANOVA followed by Dunnett post hoc tests). (d) BBB integrity to peptides **17** and **18**. Measured as permeability of fluorescent FD4 dextran upon peptide exposure. Values are mean ± SEM of *n* = 3–6. No statistical significance difference was observed between samples (one-way ANOVA followed by Tukey’s multiple comparison test). (e) Confocal microscopy. Images showing peptide internalization into bEnd.3 cells.

When **17** and **18** were submitted to the same screens as previous candidates (Figure 4), our expectations were fulfilled. The ability to interfere the CB_1_R–5HT_2A_R heterodimer structure and signaling (Figure 4a,b) was preserved, serum degradation was successfully averted (Figure S3a), and despite the extensive structural modifications undergone, BBB-crossing properties were also maintained (Figures 4c,d, S3b). This last feature was further confirmed by examining internalization of both **17** and **18** in bEnd.3 cells by confocal microscopy (Figure 4e).

### *In Vivo* Validation of Finalist Peptides **17** and **18**

The promising *in vitro* data obtained for analogues **17** and **18** encouraged us to validate their *in vivo* efficacy in mice. Given their BBB-crossing features, we evaluated the efficacy of the peptides, intravenously (IV) administered, by examining the preservation of THC analgesic effects (hot-plate test) and the disruption of its amnesic effects (novel object recognition paradigm) (see Experimental Section for a detailed description of both assays).

**Figure 5:**
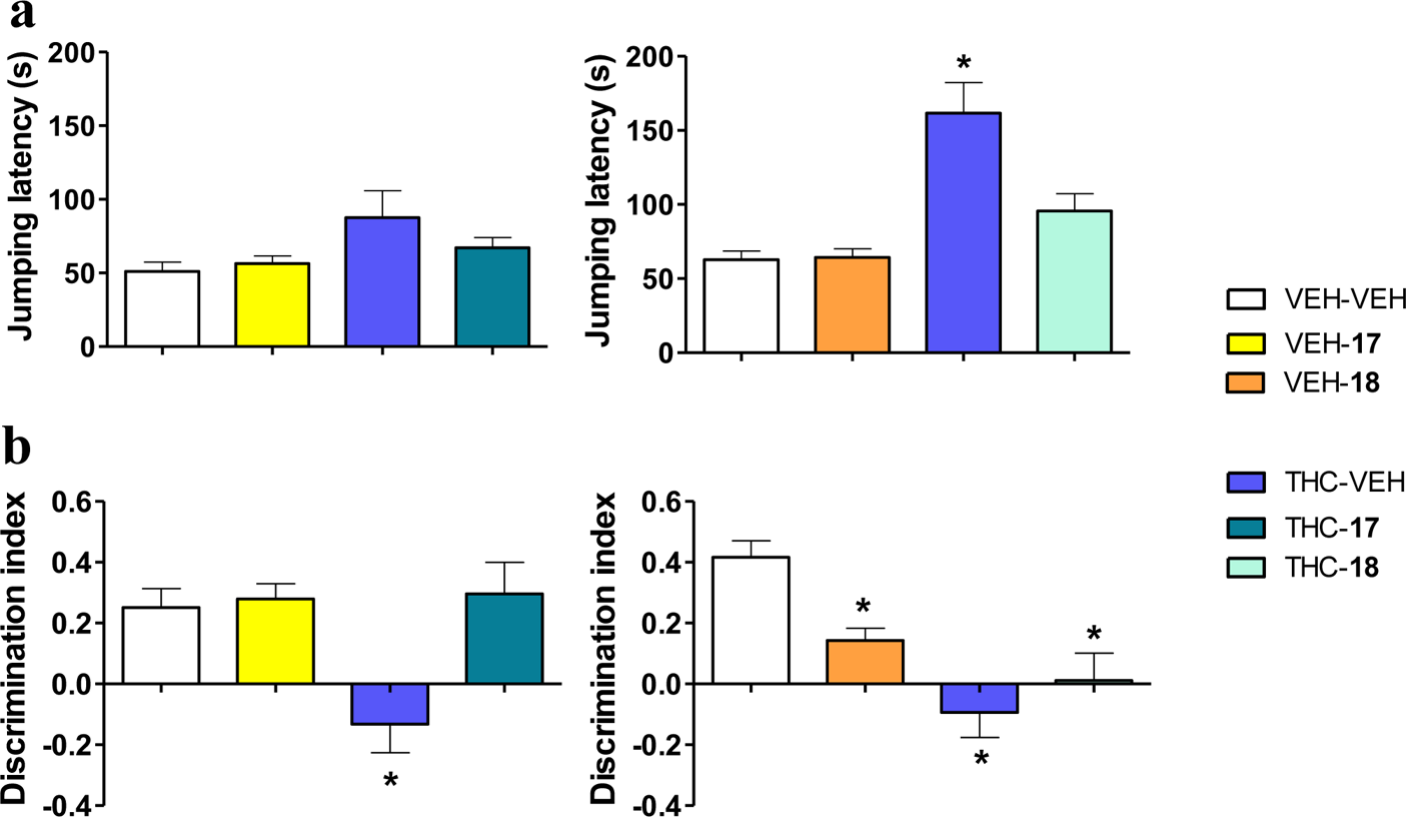
Prevention of THC-induced amnesic effects by peptide **17**, while maintaining THC analgesic responses. (a) Analgesic effects of THC (10 mg/kg, IP) in the mouse hot-plate test were preserved after pretreatment with peptides **17** and **18** (both 20 mg/kg, IV). (b) Amnesic effects of THC (3 mg/kg, IP) observed in the mouse novel object recognition test were abrogated by pretreatment with **17** (20 mg/kg, IV) but not with **18** (20 mg/kg, IV). Data are presented as mean ± SEM (*n* = 10–12). *p < 0.05 *vs* vehicle + vehicle (Fisher LSD test).

The *in vitro*-based expectations for **17** and **18** were partially fulfilled. Thus, the analgesic effects induced by THC (10 mg/kg by intraperitoneal route, IP) were preserved when coadministered with either peptide (20 mg/kg, IV) (Figure 5a). In contrast, the memory impairment produced by THC (3 mg/kg, IP) was suppressed only when coadministered with **17** (20 mg/kg, IV), but amnesic effects remained unaffected when THC was coadministered with **18** (20 mg/kg, IV) (Figure 5b).

**Figure 6:**
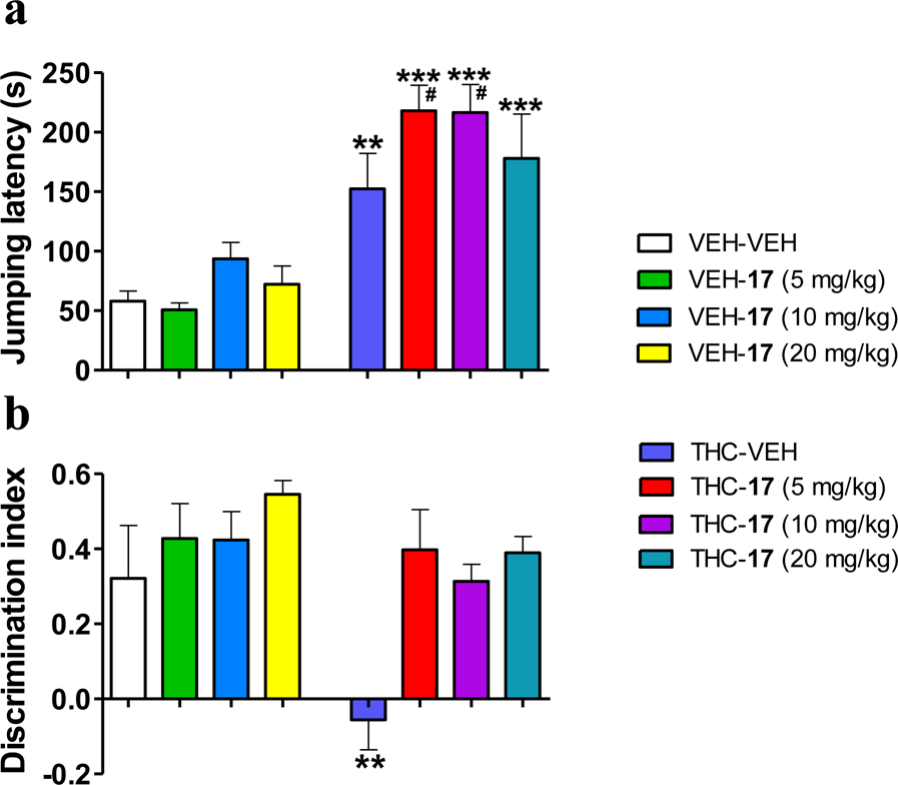
Prevention of THC-induced amnesic effects by peptide **17** administered orally, while maintaining THC analgesic responses. (a) Analgesic effects of THC (10 mg/kg, IP) revealed in the mouse hot-plate test were preserved after pretreatment with **17** (5, 10, and 20 mg/kg, OR). (b) Amnesic effects of THC (3 mg/kg, IP) observed in the mouse novel object recognition test were abrogated by pretreatment with **17** (5, 10, and 20 mg/kg, OR). Data are presented as mean ± SEM (*n* = 5–8). **p < 0.01, ***p < 0.001 *vs* vehicle + vehicle. ^#^*p* < 0.05 *vs* vehicle + THC (Fisher LSD test).

Therefore, peptide **17** was chosen to evaluate the effects following oral administration (OR), the suitable route for therapeutic purposes. THC (10 mg/kg, IP) analgesic effects remained unaffected by coadministration with 5, 10, and 20 mg/kg (OR) doses of **17** (Figure 6a), whereas amnesic effects (3 mg/kg THC, IP) were reverted by the same doses of **17** given by gavage (Figure 6b), demonstrating the effectiveness of the peptide by the most desirable route of administration.

### Non-immunogenicity Confirmed

**Figure 7:**
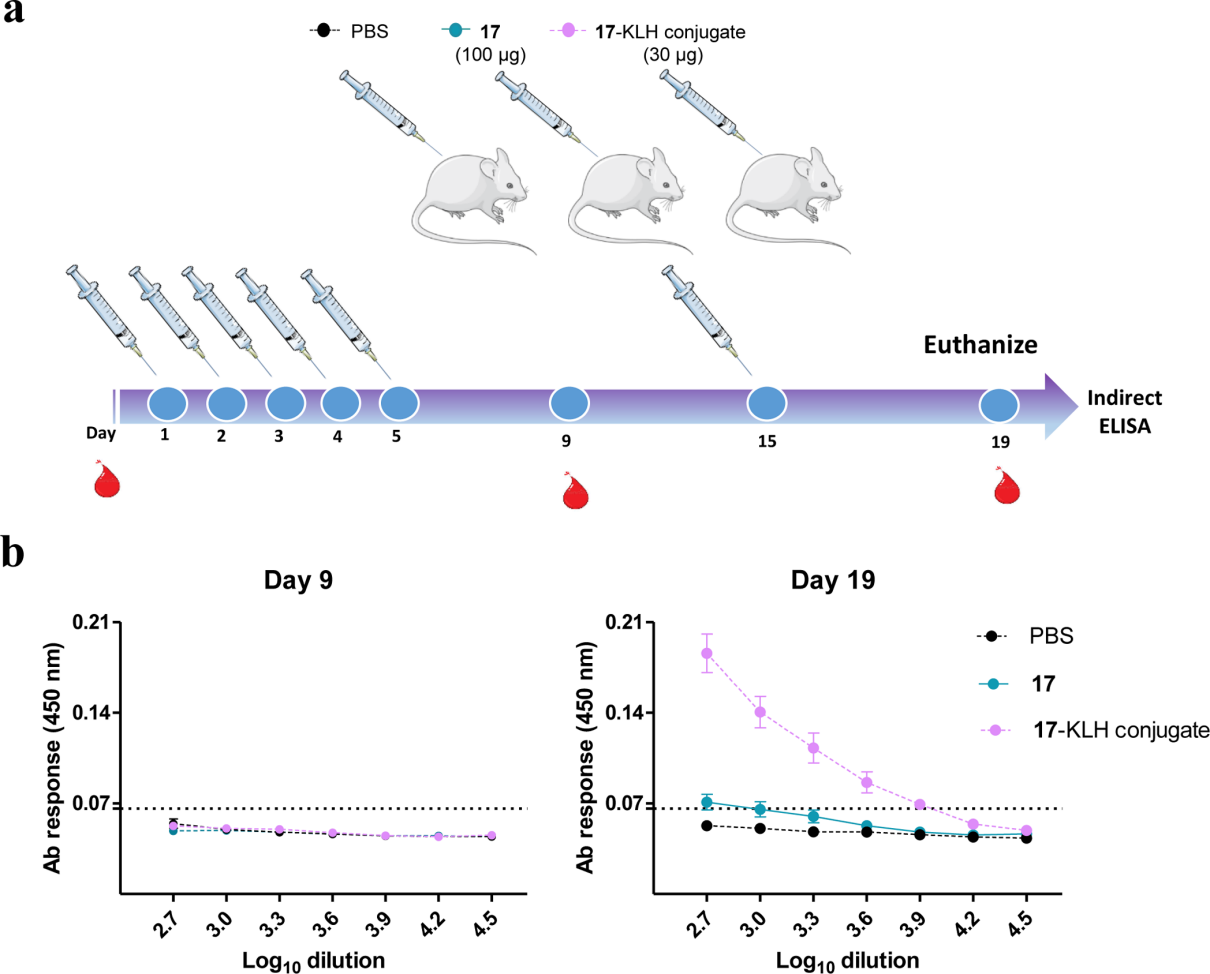
(a) Immunogenicity assessment. The procedure was devised to mimic a daily chronic pain therapy. Mice were treated for 5 consecutive days and boosted at day 15 with peptide **17** in phosphate-buffered saline (PBS) (group 1, 100 μg/dose), peptide **17**-KLH conjugate in PBS (group 2, positive control, 30 μg), or PBS (group 3, negative control). The lower dose of the **17**-KLH conjugate, compared to **17** alone, was chosen to prevent potential protein toxicity in the mice. Serum samples were collected on days 0, 9, and 19 and animals were euthanized on day 19. (b) ELISA of humoral response. Peptide-specific antibody response in sera collected at the indicated days. Each point depicts mean antibody titers ± SD. The dotted line indicates the detection limit. Free (nonconjugated) **17** induces no significant response; immunogenicity is observed only upon conjugation to a large-size carrier protein (KLH, MW ∼ 5 MDa) and boosting.

After showing that peptide **17** epitomized our wish list for an optimal *Cannabis*-complementing drug candidate, a final concern was its possible immunogenicity, despite expectations that a small 16-residue peptide would most likely go undetected by the immune system. To this end, we immunized mice using a repeated schedule with a high dosage (100 μg of **17** on 5 consecutive days, and a boost at day 15). As shown in Figure 7, repeated administration of **17** in the free form did not evoke any detectable immune response. Antibodies to **17** could only be eventually raised by immunization with the keyhole limpet hemocyanin (KLH)-conjugated version, a positive control of immunogenicity that is, on the other hand, irrelevant with regard to administration of **17** as a therapeutic agent.

In sum, our research has led to an orally active, proteolytically stable, and nonimmunogenic short peptide that constitutes an optimal candidate for combined administration with THC, taking advantage of its analgesic effects but avoiding its associated cognitive impairment.

## Discussion

CB_1_R is the most-abundant GPCR in the mammalian brain and the main target of THC.^37−40^ While originally thought to exist exclusively as a monomer,^41^ strong evidence has accumulated indicating its ability to form homo- and heterodimer complexes^42−48^ with new pharmacological and functional properties.^49^ Of particular relevance in this regard is the formation of the CB_1_R–5HT_2A_R heteromer, as previously revealed by (i) *in vitro* proximity-based biophysical techniques, (ii) specific biochemical signatures of heteromers distinct from those of the protomers, (iii) *in situ* proximity ligation assay to detect protein–protein interactions in native tissues, (iv) membrane-permeable peptides altering heteromeric interfaces, and (v) mice expressing heteromerization-deficient receptors, all of them established criteria for the existence of CB_1_R–5HT_2A_R heteromers *in vivo*.^50^ This CB_1_R–5HT_2A_R heteromer, therefore, is not simply due to receptor overexpression, but the consequence of an explicit molecular mechanism of THC to dissociate beneficial antinociceptive response and detrimental amnesic effects,^50^ hence defining it as a novel therapeutic target.

The development of heteromer-specific drugs is more challenging than traditional drug-discovery programs, where efforts are focused on compounds binding on orthosteric sites of monomeric GPCRs of endogenous ligands.^51,52^ One of the most widespread options to target heteromers is designing bivalent ligands, that is, single chemical entities capable of interacting simultaneously with the orthosteric sites of a (homo/hetero) dimer *via* two pharmacophore units covalently linked by an appropriate spacer.^53,54^ We have developed here a new approach in which the ligand/peptide binds at the TM dimerization interface of the heteromer. This rests on the assumption of allosteric binding sites at the extrahelical part of GPCRs, facing the membrane bilayer. The numerous recent structures of GPCRs bound to allosteric modulators have confirmed the presence of these sites for families A and B.^55^ Related to the GPCRs of this manuscript, the negative allosteric modulator ORG27569 of CB_1_R binds to an extrahelical site, mostly interacting with TM4.^56^

Protein–protein interfaces in general, and GPCR interfaces in particular, consist of large and flat contact areas with no well-defined pockets.^57^ Here, we have taken advantage of our previous studies in which peptides with the TM5 and TM6 sequences of CB_1_R (**TM5-Tat** and **Tat-TM6**), fused to a HIV-Tat CPP vector, can bind 5HT_2A_R and modify the quaternary structure of the CB_1_R–5HT_2A_R heteromer.^18^ MD simulations of **TM5-Tat** and **Tat-TM6** in complex with 5HT_2A_R have allowed to identify these “hotspots” near the intracellular part.

The proteolytic instability of **TM5-Tat** or **Tat-TM6** and their inability to penetrate the BBB complicate any prospect of systemic administration. With this in mind, we have set out to develop strategies aimed at improving the druggability of the **TM5-Tat** and **Tat-TM6** prototypes by (i) downsizing the length of the interfering peptides to only include the essential identified “hotspots”, (ii) enabling CNS delivery *via* a BBB-permeating CPP shuttle, (iii) ensuring proteolytic robustness, hence bioavailability, by judicious structural manipulation, including the switch to enantio and retro-enantio versions of CPP and interfering motifs, respectively. To this end, we have used *in silico* tools and peptide medicinal chemistry criteria through a series of design/optimization rounds and a variety of *in vitro* and *in vivo* screens that have eventually allowed to identify peptide wliymyayvaGilkrw (**17**) as a highly effective, orally available candidate with encouraging therapeutic prospects.

## Conclusions

Pain is a highly relevant pharmacological area with still significant unmet needs. It has been suggested that the therapeutic gap between benign but rather feeble NSAID painkillers and effective but high-risk opioids could be ideally bridged by cannabinoids, although their important undesirable side effects represent a major limitation. In addition, new markets are emerging, also aimed at using cannabis-derived products with other (i.e., non pain-related) purposes, and again entraining significant risks associated to these side effects. Our work has resulted in a novel tool to minimize the most prominent of these adverse outcomes, namely cognitive impairment. We specifically propose a new approach based on the administration of a CB_1_R cannabinoid agonist (*e.g.,* THC) in combination with a CB_1_R–5HT_2A_R altering agent. This compound is a 16-residue peptide (**17**: wliymyayvaGilkrw) that provides a convincing proof of concept that appropriately modified peptides constitute valid therapeutic candidates for treating pain with cannabinoids minimizing their side effects. This novel pharmacological approach could also be of interest for attenuating the side effects associated to the use of cannabis derivatives for other purposes.

## Experimental Section

### Computational Models

The CB_1_R–5-HT_2A_R heteromer (Figure 1a) was built from the TM5/6 dimeric interface observed in the crystal structure of the μ-opioid receptor (PDB id 4DKL),^21^ using the crystal structure of CB_1_R (5TGZ)^58^ and a 5HT_2B_-based (4IB4)^59^ homology model of 5HT_2A_R as previously proposed.^18,20^ The molecular 3D-coordinates corresponding to CB_1_R TM helices 5 and 6 were extracted and fused with the HIV-Tat(48–58) motif. The resulting **TM5-Tat** and **Tat-TM6** peptides were capped at the N- and C-termini with acetyl and carboxamide groups and energy-minimized using the Molecular Operating Environment (MOE) software (Chemical Computing Group Inc., Montreal, Quebec, Canada). Molecular complexes of 5HT_2A_R with **TM5-Tat** and **Tat-TM6** were embedded in a pre-equilibrated lipid bilayer box containing 1-palmitoyl-2-oleoyl-*sn*-glycero-3-phosphatidylcholine (POPC), water molecules (TIP3P), and monoatomic Na^+^ and Cl^–^ ions (0.2 M) (Figure 1b,c). The assignment of ionization states and hydrogens at physiological pH for the selected structures was conducted with the Protonate3D method as implemented in MOE. Molecular systems were subject to 1000 cycles of energy minimization, followed by 20 ns of gradual relaxation of positional restraints (corresponding to 100, 50, 25, and 10 kJ mol^–1^ nm^–2^) at protein backbone coordinates before the production phase in order to hydrate the receptor cavities and allow lipids to pack around the protein. The AMBER99SB force field as implemented in GROMACS and Berger parameters for POPC lipids were used for the MD simulations. After equilibration, three replicas of 1 μs of unrestrained MD simulation were performed at a constant temperature of 300 K and a time step of 2.0 fs. Lennard-Jones interactions were computed using a cutoff of 10 Å, and the electrostatic interactions were treated using PME with the same real-space cutoff under periodic boundary conditions. MD simulations were performed using GROMACS 2019.

### Peptide Synthesis, Analysis, and Purification

Peptides were assembled in a Prelude instrument (Protein Technologies, Tucson, AZ) running optimized Fmoc synthesis protocols as described before.^60^ For labeled peptides, the incorporation of 5(6)-carboxyfluorescein to the carboxy-terminal end was performed by adding an additional Fmoc-Lys(Mtt)-OH residue to the sequence, the Mtt group selectively removable on-resin with 1% trifluoroacetic acid, after which the fluorescent dye was coupled *via* its carboxyl function to the Lys free ε-NH_2_ group. Final deprotection and cleavage were performed with a CF_3_COOH/H_2_O/3,6-dioxa-1,8-octanedithiol/triisopropylsilane (94:2.5:2.5:1 v/v) cocktail for 90 min. Peptide analysis and purification were performed as previously detailed.^60^ Fractions of satisfactory purity (>90%) by analytical high-performance liquid chromatography (HPLC) were pooled, lyophilized, and analyzed for identity by HPLC–MS (Supporting Information, section 3). For immunogenicity studies, conjugation to bovine serum albumin (BSA) or KLH was carried out with a peptide containing an extra C-terminal Cys residue. Both carrier proteins were preactivated with *m*-maleimidobenzoyl-*N*-hydroxysuccinimide ester (MBS). After Sephadex G25 gel filtration, purified KLH-MBS or BSA-MBS was added to the peptide (r.t., 5 h, pH 7.0). The unreacted peptide was removed by dialysis and the peptide–protein conjugates were lyophilized and quantified by the AccQTag method.

### Enzyme Digestion

Peptide samples, incubated with trypsin, were collected and analyzed as before.^60^ For human serum digestion, peptide [1000 μM, 2% dimethyl sulfoxide (DMSO) in filtered H_2_O] was added to human serum (Sigma, St Louis, Missouri) in a 1:1 ratio and incubated at 37 °C with gentle shaking. At 0 min and 24 h, aliquots were taken and proteolysis was stopped with CH_3_CN (80% in filtered H_2_O), chilled at 0 °C for 15 min to precipitate serum proteins, and centrifuged at 13,000 rpm at 4 °C for 10 min. The supernatant was collected and analyzed by analytical RP-HPLC and LC–MS as described before,^60^ with a 0–95% linear gradient of CH_3_CN over 15 min. All experiments are performed in duplicate and data were fitted with GraphPad Prism.

### *In Vitro* Biochemical and Molecular Assays

#### Expression Vectors, HEK-293T Cell Culture, and Transient Transfection

Sequences encoding YFP Venus protein amino acid residues 1–155 and 156–238 were subcloned in the pcDNA3.1 vector to obtain the YFP Venus hemitruncated proteins. The human cDNAs for 5HT_2A_R and CB_1_R, cloned into the pcDNA3.1, were amplified and subcloned as described^18^ to give 5HT_2A_R-cYFP and CB_1_R-nYFP. Human embryonic kidney (HEK-293T) cells obtained from ATCC were grown in Dulbecco’s modified Eagle’s medium (DMEM) (Gibco) supplemented with 2 mM l-glutamine, 100 μg/mL sodium pyruvate, 100 U/mL penicillin/streptomycin, MEM nonessential amino acid solution (1/100), and 5% (v/v) heat-inactivated fetal bovine serum (FBS) (all supplements from Invitrogen, Paisley, Scotland, UK). Cells were maintained at 37 °C in an atmosphere of 5% CO_2_. HEK-293T cells were transfected with the corresponding fusion protein cDNA by the polyethylenimine (Sigma) method, as described before.^18^

#### BiFC Assay

HEK-293T cells, after 48 h transient cotransfection with the cDNA encoding for 5HT_2A_R, fused to c-YFP, and CB_1_R fused to n-YFP (4 μg of cDNA for each construct), were treated or not with the indicated peptides (4 μM) for 4 h at 37 °C. To quantify protein-reconstituted YFP Venus expression, cells (20 μg protein) were distributed in 96-well microplates (black plates with a transparent bottom, Porvair, King’s Lynn, UK), and emission fluorescence at 530 nm was read in a Fluo Star Optima fluorimeter (BMG Labtechnologies, Offenburg, Germany) equipped with a high-energy Xe flash lamp, using a 10 nm bandwidth excitation filter at 400 nm reading. Protein fluorescence expression was determined as fluorescence of the sample minus the fluorescence of cells not expressing the fusion proteins (basal). Cells expressing 5HT_2A_R-cVenus and nVenus or CB_1_R-nVenus and cVenus showed similar fluorescence levels to nontransfected cells.

#### cAMP Production and ERK-1/2 Phosphorylation Assays

For cAMP production, homogeneous time-resolved fluorescence energy transfer (HTRF) assays were performed as previously described.^18^ Cells (1000 cells/well) growing in medium containing 50 μM zardeverine were pretreated with the CB_1_R antagonist, RIM (1 μM, RIM), and the 5-HT_2A_R antagonist, MDL 100,907 (300 nM, MDL), or the corresponding vehicle in white ProxiPlate 384-well microplates (PerkinElmer, Waltham, Massachusetts, US) at 25 °C for 20 min and stimulated with the CB1 agonist, WIN 55,212-2 (100 nM, WIN) and the 5HT_2A_ agonist, DOI (100 nM, DOI) for 15 min before adding 0.5 μM FK, or vehicle and incubated for an additional 15 min period. Fluorescence at 665 nm was analyzed on a PHERAstar Flagship microplate reader equipped with an HTRF optical module (BMG Lab technologies, Offenburg, Germany).

For ERK-1/2 phosphorylation assay, HEK-293 cells (30.000 cells/well) seeded in 96-well poly-d-lysine-coated plates (Sigma-Aldrich, Madrid, Spain) were pretreated at 25 °C for 15 min with RIM (1 μM, RIM) and MDL 100,907 (300 nM, MDL) or the corresponding vehicle and stimulated for an additional 7 min with the WIN 55,212-2 (100 nM, WIN) and DOI (100 nM, DOI). Phosphorylation was determined in white ProxiPlate 384-well microplates (PerkinElmer Life Sciences) by the α-screen bead-based technology using the amplified luminescent proximity homogeneous assay kit (PerkinElmer Life Sciences) and using the Enspire multimode plate reader (PerkinElmer Life Sciences). Phosphorylation is expressed in arbitrary units, ALPHAcounts, as measured by light emission at 520–620 nm of the acceptor beads.

#### bEnd.3 Cell Culture and Viability Assay

bEnd.3 brain endothelial cells (ATCC CRL-2299TM) were grown as a monolayer in DMEM supplemented with 10% FBS and 1% penicillin/streptomycin solution (Gibco, USA). Cells were cultured in a humidified atmosphere of 95% air and 5% CO_2_ at 37 °C (MCO-18AIC (UV), Sanyo, Japan), with daily medium replacement.

The cytotoxicity of peptides toward bEnd.3 cells seeded into a 96-well plate (Corning, USA) at 10,000 cells/100 μL/well was tested using CellTiter-Blue cell viability assay.^31^ IC_50_ values were determined with GraphPad Prism using a log(inhibitor) *vs* normalized response. Experiments were performed on different days using independently grown cell cultures.

### *In Vitro* BBB Translocation and Integrity Studies

The translocation capabilities of 5(6)-carboxyfluorescein-labeled peptides were determined using a protocol previously described.^30,31^ After the experiment, the integrity of the endothelial barrier was checked by measuring the permeability of a 4 kDa fluorescently labeled dextran (FD4) as previously described.^25^ Experiments were carried out on different days using independently grown cell cultures.

### Confocal Microscopy

The internalization of 5(6)-carboxyfluorescein-labeled peptides was evaluated using confocal microscopy as previously described.^31^ The acquisition was done on a confocal point-scanning Zeiss LSM 880 microscope (Carl Zeiss, Germany) equipped with an alpha Plan-Apochromat X 63 oil immersion objective (1.40 numerical aperture). A diode 405–30 laser was used to excite Hoechst 33342 (Sigma-Aldrich, Spain) and CellMask Deep Red plasma membrane stain (Thermo Fisher, USA). The 488 nm line from an Ar laser was used to excite labeled peptides. In the normal confocal mode, X 0.6 zoom images were recorded at 1024 × 1024 resolution. ZEN software was used for image acquisition and Fiji software for image processing.

### *In Vivo* Behavior and Pain Response Assays

#### Memory Impairment Measurements

On day 1 (Habituation), mice were habituated for 9 min to the V-maze in which the task was performed. The following day (Training), mice were placed in the V-maze for 9 min, two identical objects were presented and the time that the mice spent exploring each object was recorded. Mice were again placed in the maze 24 h later for 9 min (Test), one of the familiar objects was replaced with a novel object and the total time spent exploring each of the two objects (novel and familiar) was recorded. Object exploration was defined as the orientation of the nose to the object at less than 2 cm. A discrimination index was calculated as the difference between the time spent exploring the novel or familiar object divided by the total time exploring the two objects. A higher discrimination index is considered to reflect greater memory retention for the familiar object.

Peptide or vehicle were administered by IV (20 mg/kg) or oral route (5, 10, and 20 mg/kg) immediately after the training session. Thirty min after peptide administration, mice received an IP injection of vehicle (5% v/v of ethanol, 5% v/v of Cremophor-EL, and 90% v/v saline) or 3 mg/kg of THC dissolved in vehicle.

#### Hot-Plate Test

THC-induced analgesia was measured using a hot-plate meter (Hot/Cold Plate Test, Bioseb, USA) 60 min after IP injection of either THC dissolved in IP vehicle or vehicle alone. Peptide or vehicle were administered by IV (20 mg/kg) or oral route (5, 10 and 20 mg/kg) 30 min before THC. The plate was heated to 52 ± 0.5 °C and the time (in s) until mice showed a jumping response on the plate was recorded. A cutoff time of 240 s was set to prevent tissue damage.

#### Mice Immunization, Bleedings, and Serum Analysis by ELISA

Experiments were performed at the ICTS “NANBIOSIS” Custom Antibody Service (CAbS, CIBER-BBN, IQAC-CSIC). The immune response to **17** was assessed in outbred Swiss ICR (CD-1) mice strain Hsd:ICR (CD-1) (Envigo RMS, The Netherlands), following the immunization schedule in Figure 7. Animals were housed under standard conditions in the CID-CSIC animal facility. Animal experimental procedures were conducted in accordance with protocols approved by CSIC Committee on Ethics of Animal Experiments and Biosafety, as well as the Spanish National Committee on Ethics and Animal Welfare. Briefly, a first group of 10 female mice received IV doses of **17** (200 μL, 20 mg/kg, PBS + 2% DMSO), a second group of 5 mice received IV doses of **17**-KLH conjugate (200 μL, 30 μg) and a third group of 5 mice received IVdoses of PBS (200 μL, negative control). The **17**-KLH conjugate, administered with Freund’s adjuvant, was also used to generate polyclonal antibody (positive control) (data not shown). Serum samples were collected by tail vein bleeds on days 0, 9, and 19. The animals were humanely sacrificed on day 19. The peptide-specific humoral response was determined by indirect ELISA. Microtiter plates (Nunc) were coated with the **17**-BSA conjugate (1 μg/mL in coating buffer, 100 μL/well) overnight at 4 °C. The next day, plates were washed and antiserum samples (diluted in PBST, serial dilutions starting at 1/100, 100 μL/well) were added and incubated for 30 min at r.t.. The plates were again washed and a solution of anti-IgG–HRP (1/4000 in 10 mM PBST) was added to the wells (100 μL/well) and incubated for 30 min at r.t.. After another wash, the tetramethylbenzidine substrate solution was added (100 μL/well) to plates. Color development was stopped with 4 N H_2_SO_4_ (50 μL/well) after 30 min at r.t., and absorbance read at 450 nm. Titers in a log_10_ scale were expressed as the reciprocal of the last dilution giving the absorbance recorded in the control wells (serum at day 0) plus 2 SD.

#### Statistical Analysis

Experimental data were managed and analyzed with GraphPad Prism software version 9 (San Diego, CA, USA) or IBM SPSS Statistics version 27.0 (IBM Corp., NY, USA). *P*-values lower than 0.05 were considered statistically significant.

## Supporting Information Available

Design and synthesis of TMs 5 and 6 short analogues; HIV-Tat replacement by shorter CPPs; HIV-Tat replacement by shorter CPPs; applying CPP stereochemistry change; applying configurational switch and sequence reversal for last-stage lead optimization; statistical analyses used in signaling experiments; stabilities of the peptide-receptor complexes; effect of CPP stereochemistry on trypsin digestion; HPLC analysis of serum-incubated peptides 17 and 18; analytical data; and molecular formula strings (PDF)
Crystal structure of the μ-opioid receptor (PDB)
Molecular formula strings (CSV)

## Supplementary Material

jm1c00484_si_001.pdf

jm1c00484_si_002.pdb

## Notes

Authors will release the atomic coordinates upon article publication.

## Abbreviations

BiFC: bimolecular fluorescence complementation
BBB: blood–brain barrier
CB_1_R: cannabinoid receptor 1
CNS: central nervous system
CPP: cell-penetrating peptide
DOI: 2,5-dimethoxy-4-iodoamphetamine
FK: forskolin
GPCR: G-protein-coupled receptor
ICV: intracerebroventricular
IP: intraperitoneal
IV: intravenous
KLH: keyhole limpet hemocyanin
MD: molecular dynamics
MDL: MDL 100,907
NSAID: nonsteroidal anti-inflammatory drug
r/e: retro-enantio
5HT_2A_R: serotonin (5-hydroxytryptamine, 5-HT) receptor 2A
THC: tetrahydrocannabinol
TM: transmembrane
WIN: WIN 55,212-2
YFP: yellow fluorescent protein
