# Supplementary material for: Orally Active Peptide Vector Allows Using Cannabis to Fight Pain While Avoiding Side Effects
Source: J Med Chem. 2021 Apr 23;64(10):6937–48. doi: 10.1021/acs.jmedchem.1c00484 (PMC8486167; doi:10.1021/acs.jmedchem.1c00484)
Supplement: Supplementary file 1 — jm1c00484_si_001.pdf [file jm1c00484_si_001.pdf]

Supporting Information

An orally active peptide vector allows using cannabis to fight pain while avoiding side effects

Maria Gallo,[†,#] Estefanía Moreno,[‡,#] Sira Defaus,[†] Antonio Ortega-Alvaro,[†] Angel Gonzalez,[§] Patricia Robledo,[^] Marco Cavaco,[‖] Vera Neves,[‖] Miguel A. R. B. Castanho,[‖] Vicent Casadó,[‡] Leonardo Pardo,[§] Rafael Maldonado,[*,†] David Andreu[*,†]

[†]Department of Experimental and Health Sciences, Universitat Pompeu Fabra, Barcelona Biomedical Research Park, 08003 Barcelona, Spain.
[‡]Department of Biochemistry and Molecular Biomedicine, Institute of Biomedicine, University of Barcelona, 08028 Barcelona, Spain.
[§]Laboratori de Medicina Computacional, Unitat de Bioestadística, Facultat de Medicina, Universitat Autònoma de Barcelona, 08193 Bellaterra, Spain.
[^]Integrative Pharmacology and Systems Neuroscience, IMIM-Hospital del Mar Research Institute, 08003 Barcelona, Spain.
[‖]Instituto de Medicina Molecular, Faculdade de Medicina, Universidade de Lisboa, 1649-028 Lisboa, Portugal.

* Rafael Maldonado, Phone: (+34) 933160824, rafael.maldonado@upf.edu
* David Andreu, Phone: (+34) 933160868, david.andreu@upf.edu

Contents

	Pages
**1. Supporting Information Tables**	
Table S1	S4
Table S2	S5
Table S3	S6
Table S4	S7
Table S5	S8
**2. Supporting Information Figures**	
Figure S1	S10
Figure S2	S11
Figure S3	S12
**3. Analytical data**	
HPLC-MS analyses	S14-S19
**4. Molecular Formula Strings**	
SMILES notations	S21-S25

1. Supporting Information Tables

Table S1. Design and synthesis of TMs 5 and 6 short analogs

Peptide	Sequence[a,b]	Theoretical mass (Da)	Experimental mass (Da)	HPLC gradient[c] (% ACN)	HPLC retention time (min)
**TM5-Tat**	ETYLMFWIGVTSVLLLFIVYAYMYILWGRKKRRQRRR	4726.8	4725.8	40-90 %	6.86
**1**	VYAYMYILWGRKKRRQRRR	2599.2	2598.7	10-50 %	5.99
**2**	VYAYMYILW	1220.5	1219.4	25-60%	7.75
**Tat-TM6**	GRKKRRQRRRKTLVLILVVLIICWGPLLAIMVYDVF	4322.5	4322.6	0-95 %	10.75
**3**	GRKKRRQRRRKTLVLILVV	2375.0	2375.1	10-60 %	5.81
**4**	KTLVLILVV	996.3	995.7	20-60%	9.50
**TM7-Tat**	LIKTVFAFCSMLCLLNSTVNPIIYALRGRKKRRQRRR	4420.4	4421.4	30-60 %	5.87

[a] All peptides are *C*-terminal carboxamides; [b] Tat(48-57) sequence GRKKRRQRRR; [c] linear gradient over 15 min, initial and final ACN concentrations noted.

Table S2. HIV-Tat replacement by shorter CPPs

Peptide	Sequence[a,b,c]	Theoretical mass (Da)	Experimental mass (Da)	HPLC gradient[d] (% ACN)	HPLC retention time (min)
**1**	VYAYMYILWGRKKRRQRRR	2599.2	2598.7	10-50	5.99
**5**	VYAYMYILWAGILKRW	2045.5	2045.4	25-50	8.83
**6**	VYAYMYILWSKSKSK	1866.3	1866.1	15-50	8.19
**7**	VYAYMYILWRLRWR	1988.4	1988.1	25-55	6.57
**8**	VYAYMYILWRKR	1661.0	1661.5	20-45	6.09
**3**	GRKKRRQRRRKTLVLILVV	2375.0	2375.1	10-60	5.81
**9**	AGILKRWKTLVLILVV	1821.4	1820.9	25-55	7.10
**10**	SKSKSKKTLVLILVV	1642.1	1642.7	15-60	7.43
**11**	RLRWRKTLVLILVV	1764.3	1764.2	15-70	8.25
**12**	RKRKTLVLILVV	1436.9	1436.6	20-45	6.01

[a] All peptides are *C*-terminal carboxamides; [b] residues in red correspond to Tat(48-57); [c] residues in blue correspond to various shorter CPPs (see main text); [d] linear gradient over 15 min, initial and final ACN concentrations noted.

Table S3. Applying CPP stereochemistry change

Peptide	Sequence[a,b]	Theoretical mass (Da)	Experimental mass (Da)	HPLC gradient[c] (% ACN)	HPLC retention time (min)
**5**	VYAYMYILWAGILKRW	2045.5	2045.4	25-50	8.83
**6**	VYAYMYILWSKSKSK	1866.3	1866.1	15-50	8.19
**13**	VYAYMYILWaGilkrw	2045.5	2045.3	30-60	4.89
**14**	VYAYMYILWsksksk	1866.3	1867.5	15-50	8.01
**9**	AGILKRWKTLVLILVV	1821,4	1820.9	25-55	7.10
**10**	SKSKSKKTLVLILVV	1642.1	1642.1	15-60	7.43
**15**	aGilkrwKTLVLILVV	1821.4	1821.2	25-50	5.90
**16**	skskskKTLVLILVV	1642.1	1642.8	15-60	7.17

[a] All peptides are *C*-terminal carboxamides; [b] residues in blue correspond to the *in vitro* optimally performing L- amino acid CPPs (see Figure S2); in magenta to the D-CPP counterparts; [c] linear gradient over 15 min, initial and final ACN concentrations noted.

Table S4. Applying configurational switch and sequence reversal for last-stage lead optimization

Peptide	Sequence[a,b,c]	Theoretical mass (Da)	Experimental mass (Da)	HPLC gradient[d] (% ACN)	HPLC retention time (min)
**13**	VYAYMYILWaGilkrw	2045.5	2045.3	30-60	4.89
**17**	wliymyayvaGilkrw	2045.5	2045.3	30-55	6.93
**15**	aGilkrwKTLVLILVV	1821.4	1821.2	25-50	5.90
**18**	aGilkrwvvlilvltk	1821.4	1821.2	30-60	6.14

[a] All peptides are *C*-terminal carboxamides; [b] residues in magenta correspond to the *in vitro* optimally performing CPP/BBB shuttle (see Figure S2-S3); [c] upper and lowercase denote L- and D-residues, respectively; [d] linear gradient over 15 min, initial and final ACN concentrations noted.

Table S5. Statistical analyses used in signaling experiments

<table>
<tr><th></th><th>Factor</th><th>p-value</th><th>Conclusions</th></tr>
<tr><td>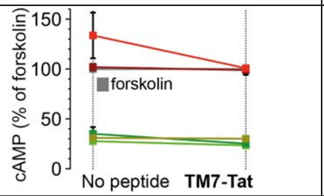
</td><td>ligand
peptide
interaction</td><td><0.0001
0.049
0.218</td><td>$5HT_{2A}$Rago < FK (p<0.0001)
CB_1Rago < FK (p<0.0001)
$5HT_{2A}$Rago+CB_1Rago < $5HT_{2A}$Rago (p=0.981)
$5HT_{2A}$Rago+CB_1Rago < CB_1Rago (p>0.999)
$5HT_{2A}$Rago+CB_1Ranta vs. $5HT_{2A}$Rago (p<0.0001)
CB_1Rago+$5HT_{2A}$Ranta vs. CB_1Rago (p<0.0001)</td></tr>
<tr><td>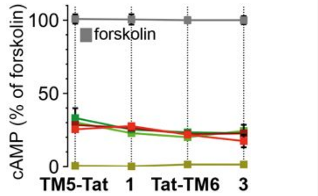
</td><td>ligand
peptide
interaction</td><td><0.0001
0.004
0.297</td><td>$5HT_{2A}$Rago < FK (p<0.0001)
CB_1Rago < FK (p<0.0001)
$5HT_{2A}$Rago+CB_1Rago < $5HT_{2A}$Rago (p<0.0001)
$5HT_{2A}$Rago+CB_1Rago < CB_1Rago (p<0.0001)
$5HT_{2A}$Rago+CB_1Ranta vs. $5HT_{2A}$Rago (p=0.99)
CB_1Rago+$5HT_{2A}$Ranta vs. CB_1Rago (p=0.49)
TM5-Tat vs. **1** (p=0.200)
Tat-TM6 vs. **3** (p>0.999)</td></tr>
<tr><td>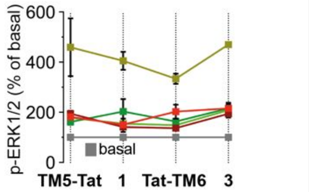
</td><td>ligand
peptide
interaction</td><td><0.0001
0.018
0.605</td><td>$5HT_{2A}$Rago > basal (p=0.007)
CB_1Rago > basal (p=0.001)
$5HT_{2A}$Rago+CB_1Rago > $5HT_{2A}$Rago (p<0.0001)
$5HT_{2A}$Rago+CB_1Rago > CB_1Rago (p<0.0001)
$5HT_{2A}$Rago+CB_1Ranta vs. $5HT_{2A}$Rago (p=0.999)
CB_1Rago+$5HT_{2A}$Ranta vs. CB_1Rago (p=0.922)
TM5-Tat vs. **1** (p=0.661)
Tat-TM6 vs. **3** (p=0.02)</td></tr>
<tr><td>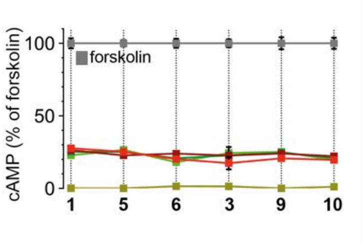
</td><td>ligand
peptide
interaction</td><td><0.0001
0.04
0.412</td><td>$5HT_{2A}$Rago < FK (p<0.0001)
CB_1Rago < FK (p<0.0001)
$5HT_{2A}$Rago+CB_1Rago < $5HT_{2A}$Rago (p<0.0001)
$5HT_{2A}$Rago+CB_1Rago < CB_1Rago (p<0.0001)
$5HT_{2A}$Rago+CB_1Ranta vs. $5HT_{2A}$Rago (p=0.975)
CB_1Rago+$5HT_{2A}$Ranta vs. CB_1Rago (p=0.821)
1 vs. **5** (p=0.999)
1 vs. **6** (p=0.141)
3 vs. **9** (p=0.958)
3 vs. **10** (p=0.981)</td></tr>
<tr><td>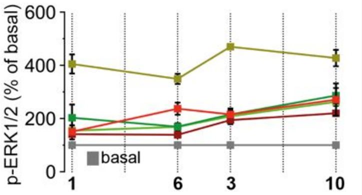
</td><td>ligand
peptide
interaction</td><td><0.0001
<0.0001
0.061</td><td>$5HT_{2A}$Rago > basal (p<0.0001)
CB_1Rago > basal (p<0.0001)
$5HT_{2A}$Rago+CB_1Rago > $5HT_{2A}$Rago (p<0.0001)
$5HT_{2A}$Rago+CB_1Rago > CB_1Rago (p<0.0001)
$5HT_{2A}$Rago+CB_1Ranta vs. $5HT_{2A}$Rago (p=0.658)
CB_1Rago+$5HT_{2A}$Ranta vs. CB_1Rago (p>0.999)
1 vs. **6** (p=0.999)
3 vs. **10** (p=0.192)</td></tr>
<tr><td>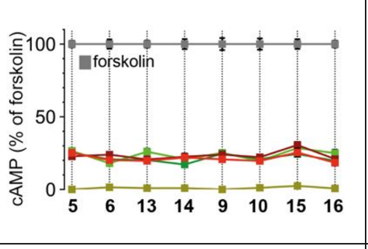
</td><td>ligand
peptide
interaction</td><td><0.0001
<0.0001
0.095</td><td>$5HT_{2A}$Rago < FK (p<0.0001)
CB_1Rago < FK (p<0.0001)
$5HT_{2A}$Rago+CB_1Rago < $5HT_{2A}$Rago (p<0.0001)
$5HT_{2A}$Rago+CB_1Rago < CB_1Rago (p<0.0001)
$5HT_{2A}$Rago+CB_1Ranta vs. $5HT_{2A}$Rago (p=0.999)
CB_1Rago+$5HT_{2A}$Ranta vs. CB_1Rago (p=0.999)
5 vs. **13** (p=0.497)
6 vs. **14** (p>0.999)
9 vs. **15** (p=0.131)
10 vs. **16** (p>0.999)</td></tr>
<tr><td>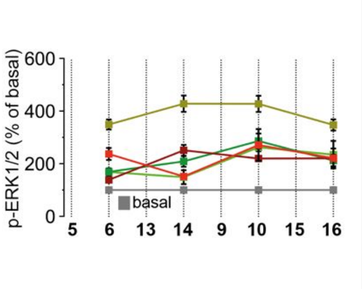
</td><td>ligand
peptide
interaction</td><td><0.0001
0.002
0.007</td><td>$5HT_{2A}$Rago > basal (**6**:p=0.19;**14**:p=0.37;**10**:p=0.0001;**16**:p=0.002)
CB_1Rago > basal (**6**:p=0.18;**14**:p=0.02;**10**:p<0.0001;**16**:p=0.01)
$5HT_{2A}$Rago+CB_1Rago > $5HT_{2A}$Rago (**6**,**14**,**10**:p<0.001;**16**:p=0.01)
$5HT_{2A}$Rago+CB_1Rago > CB_1Rago (**6**,**14**,**10**:p<0.001;**16**:p=0.002)
$5HT_{2A}$Rago+CB_1Ranta vs. $5HT_{2A}$Rago (**6**,**10**,**16**:p>0.8;**14**:p=0.053)
CB_1Rago+$5HT_{2A}$Ranta vs. CB_1Rago (**6**,**14**, **16**,**10**:p>0.58)
6 vs. **14** ($5HT_{2A}$Rago:p=0.94; CB_1Rago:p=0.67;
$5HT_{2A}$Rago+CB_1Rago:p=0.12; $5HT_{2A}$Rago+CB_1Ranta:p=0.01;
CB_1Rago+$5HT_{2A}$Ranta:p=0.08)
10 vs. **16** ($5HT_{2A}$Rago:p=0.85; CB_1Rago:p=0.15;
$5HT_{2A}$Rago+CB_1Rago:p=0.11; $5HT_{2A}$Rago+CB_1Ranta:p>0.999;
CB_1Rago+$5HT_{2A}$Ranta:p=0.47)</td></tr>
<tr><td>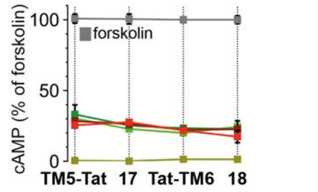
</td><td>ligand
peptide
interaction</td><td><0.0001
<0.0001
0.057</td><td>$5HT_{2A}$Rago < FK (p<0.0001)
CB_1Rago < FK (p<0.0001)
$5HT_{2A}$Rago+CB_1Rago < $5HT_{2A}$Rago (p<0.0001)
$5HT_{2A}$Rago+CB_1Rago < CB_1Rago (p<0.0001)
$5HT_{2A}$Rago+CB_1Ranta vs. $5HT_{2A}$Rago (p=0.828)
CB_1Rago+$5HT_{2A}$Ranta vs. CB_1Rago (p=0.594)
TM5-Tat vs. **17** (p<0.0001)
Tat-TM6 vs. **18** (p=0.432)</td></tr>
</table>

Two-way ANOVA followed by Tukey's multiple comparison tests was used to analyze the data depicted in the left column.

2. Supporting Information Figures

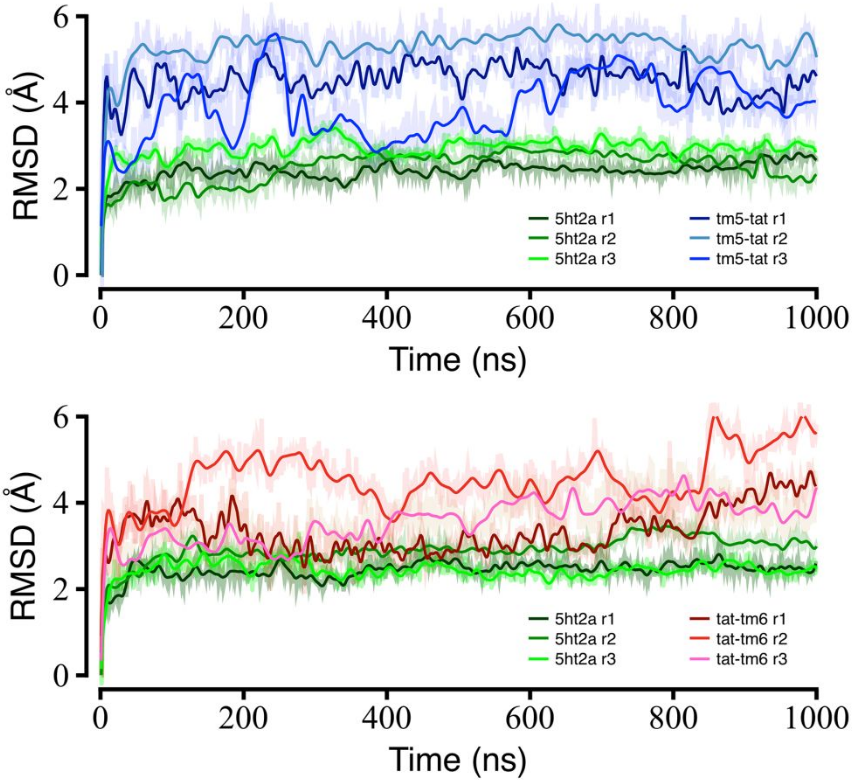

Figure S1. The stabilities of the peptide-receptor complexes, during the three replicas (r1, r2, r3) of unbiased MD simulations, are analyzed via root mean-square deviations (RMSD) of the $5HT_{2A}R$ backbone atoms (in green), **TM5-Tat** (in blue) and **Tat-TM6** (in red) peptides, derived from CB_1R. As expected, rmsd values of the peptides are larger than those of the $5HT_{2A}R$ backbone atoms, largely due to the flexibility of the Tat sequence in the water environment.

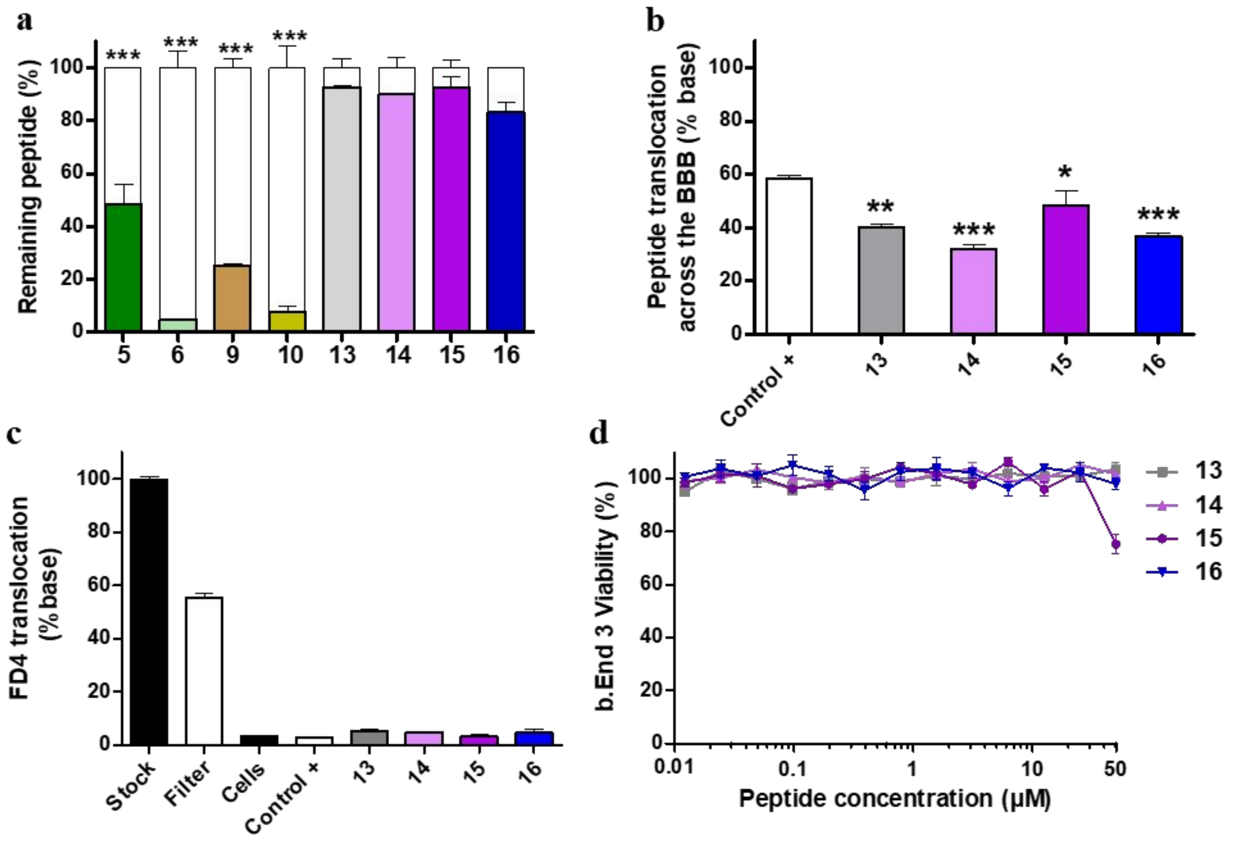

Figure S2. a, Effect of CPP stereochemistry on trypsin digestion. Unaltered peptide (color-filled bar) remaining after 24 h trypsin incubation. Main peak integrated areas at 0 h denote 100% undigested peptide (empty bars). The data are presented as mean ± SEM of n=2. One-way ANOVA followed by a Dunnett post hoc test showed a significant poor survival for **5**, **6**, **9**, **10** after 24 h vs 0 h ($^{***}p < 0.001$). **b, *In vitro* translocation of peptides 13-16.** Translocation (%) across the transwell BBB model quantified as fluorescence in the basolateral chamber after 24 h. A peptide with trans-BBB property was used as positive control. Values are mean ± SEM of n= 3–6. * $p < 0.05$, ** $p < 0.01$, *** $p < 0.001$ vs positive control (One-way ANOVA followed by a Dunnett post hoc tests). **c, BBB integrity to peptides 13-16.** Measured as permeability of fluorescent FD4 dextran upon peptide exposure. Values are mean ± SEM of n= 3–6. Statistical significance analysis was evaluated with a one-way ANOVA followed by Tukey's multiple comparison test and no statistical significance difference was observed between samples. **d, *In vitro* cytotoxicity of 13-16 towards bEnd.3 cells.** Viability (%) upon peptide incubation (0.01 – 50 μM) determined by CellTiter-Blue® Assay. Values are mean ± SEM of n= 3–6.

a

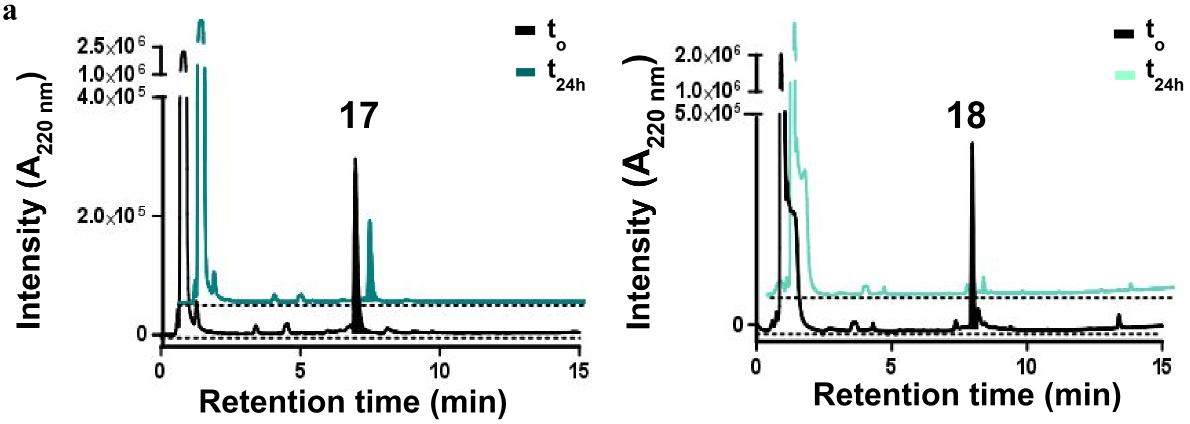

b

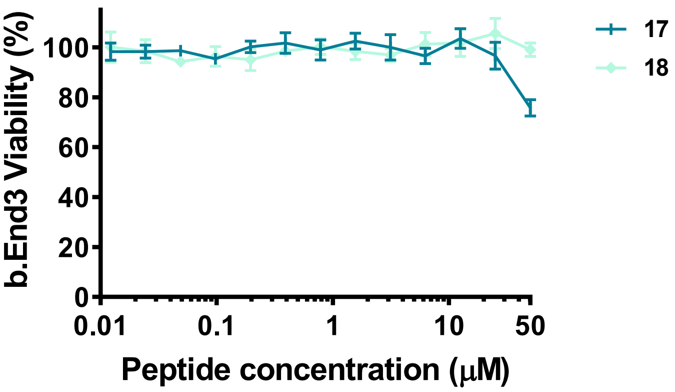

Figure S3. a, HPLC analysis of serum-incubated peptides 17 and 18. No significant new peaks referring to digested fragments were identified after 24 h. Peptide peak area decrease at 24h could be presumably attributed to binding to serum proteins. **b, *In vitro* cytotoxicity of 17 and 18 towards bEnd.3 cells.** Viability (%) upon peptide incubation (0.01 – 50 µM) determined by CellTiter-Blue® Assay. Values are mean ± SEM of n= 3–6.

3. Analytical data

HPLC-MS analysis. Left: Overlaid HPLC traces of crude (grey) and purified peptide products (blue). Right: ESI-MS spectra of pure peptides.

TM5-Tat: ETYLMFWIGVTSVLLLFIVYAYMYILWGRKKRRQRRR

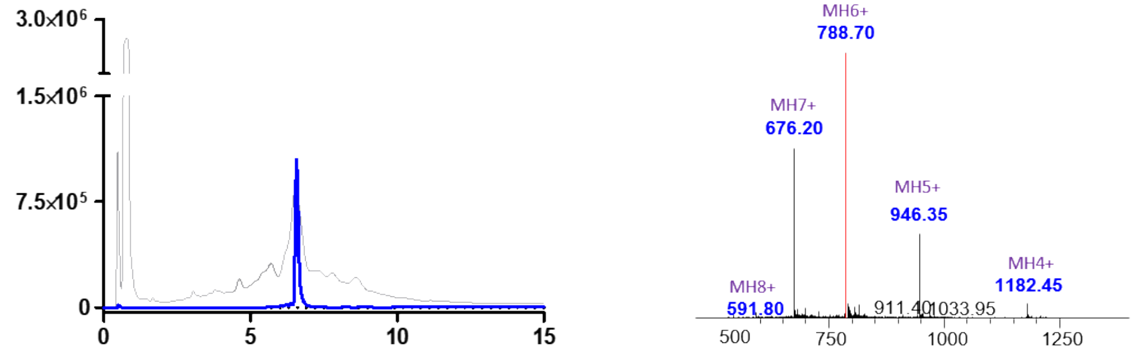

Tat-TM6: GRKKRRQRRRKTLVLILVVLIICWGPLLAIMVYDVF

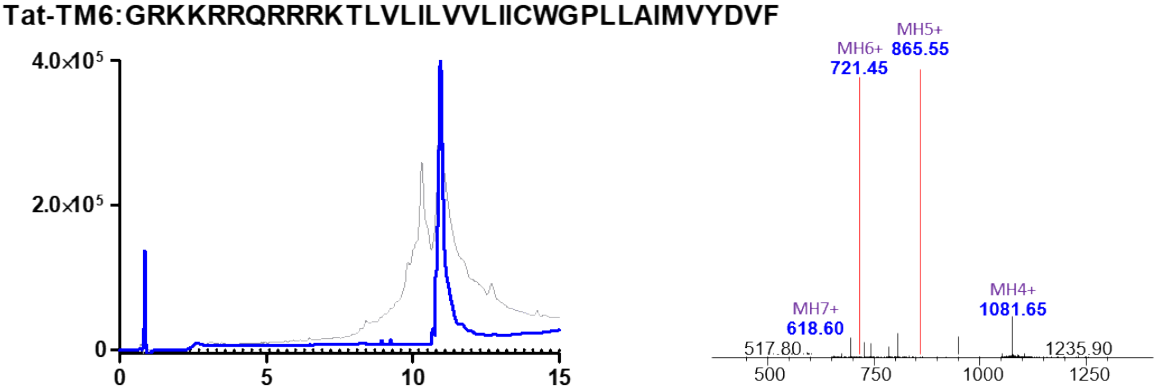

TM7-Tat: LIKTVFAFCSMLCLLNSTVNPIIYALRGRKKRRQRRR

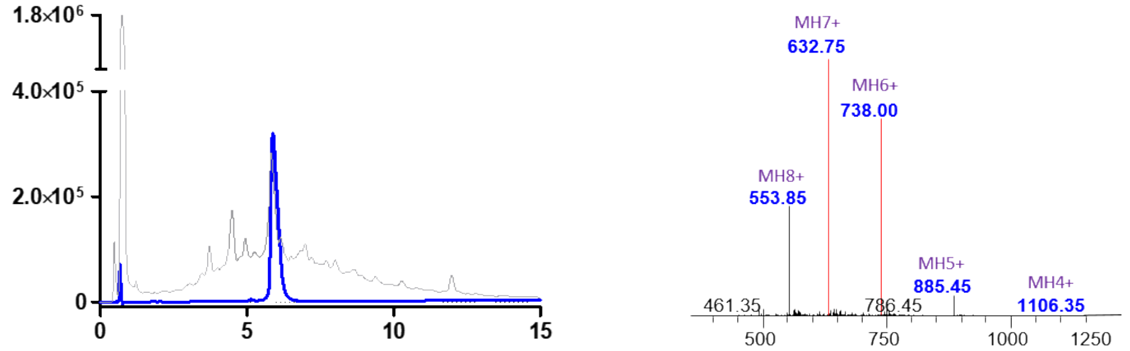

1: VYAYMYILWGRKKRRQRRR

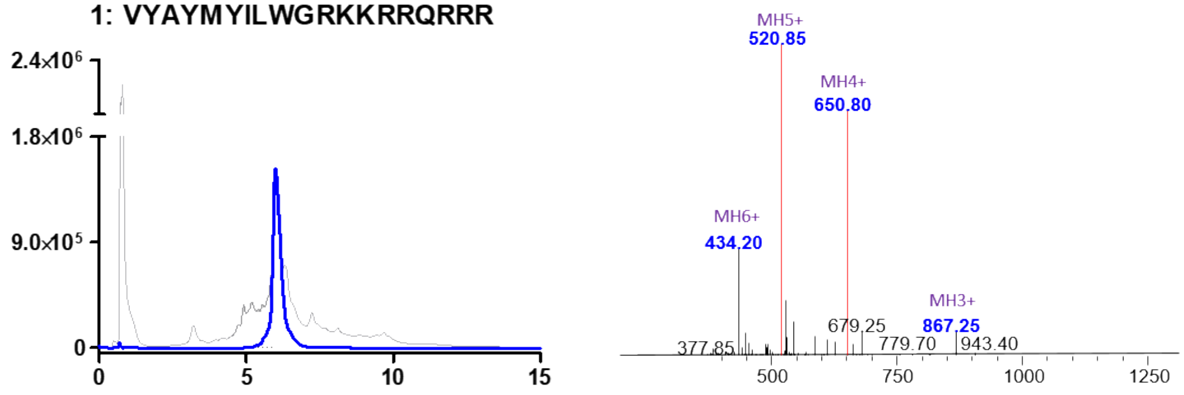

2: VYAYMYILW

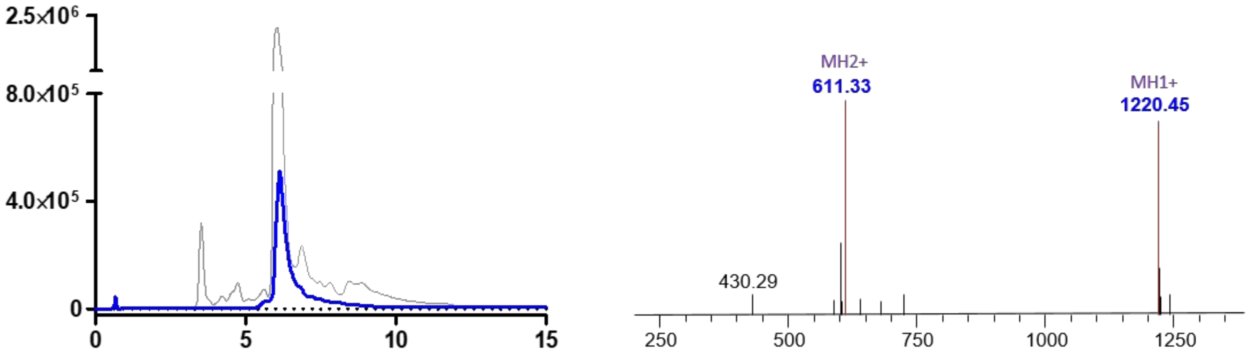

3: GRKKRRQRRRKTLVLILVV

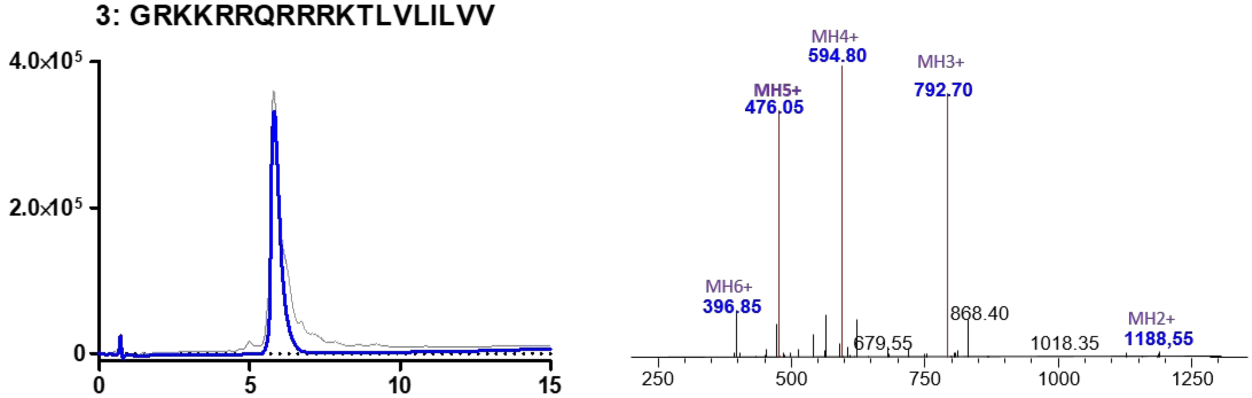

4: KTLVLILVV

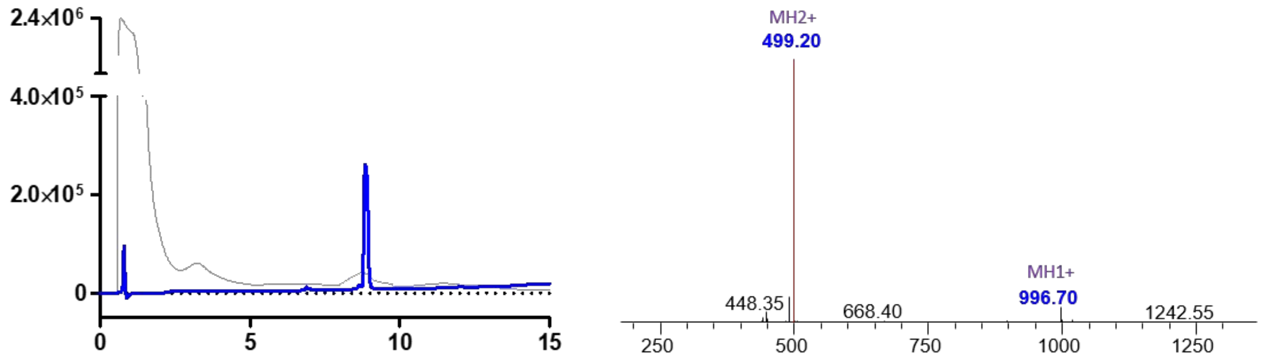

5: VYAYMYILWAGILKRW

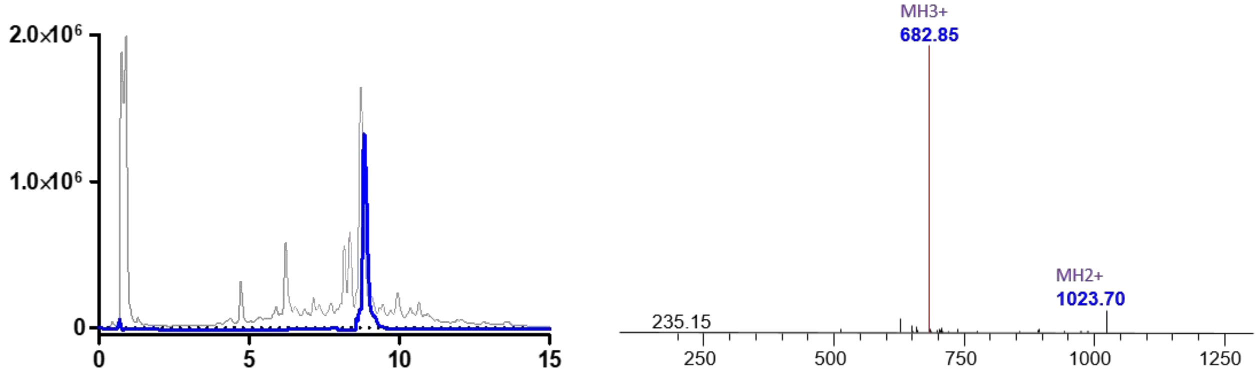

6: VYAYMYILWSKSKSK

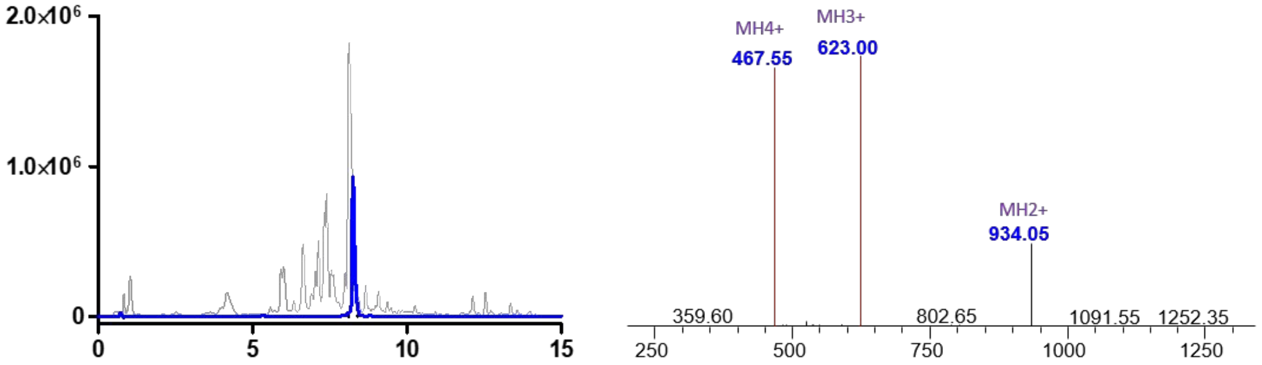

7: VYAYMYILWRLRWR

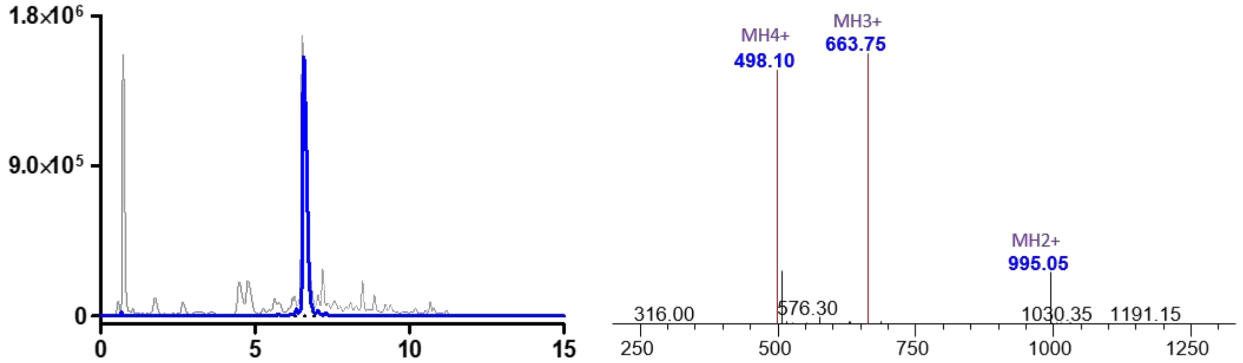

8: VYAYMYILWRKR

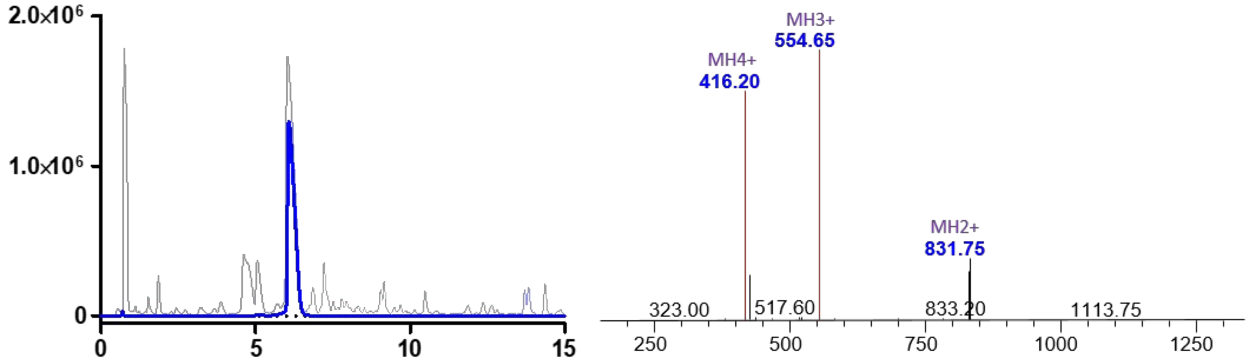

9: AGILKRWKTLVLILVV

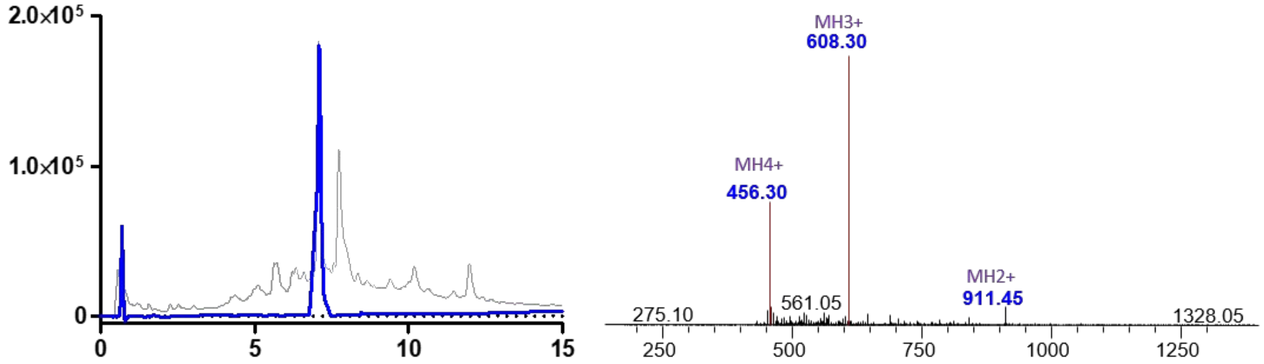

10: SKSKSKKTLVLILVV

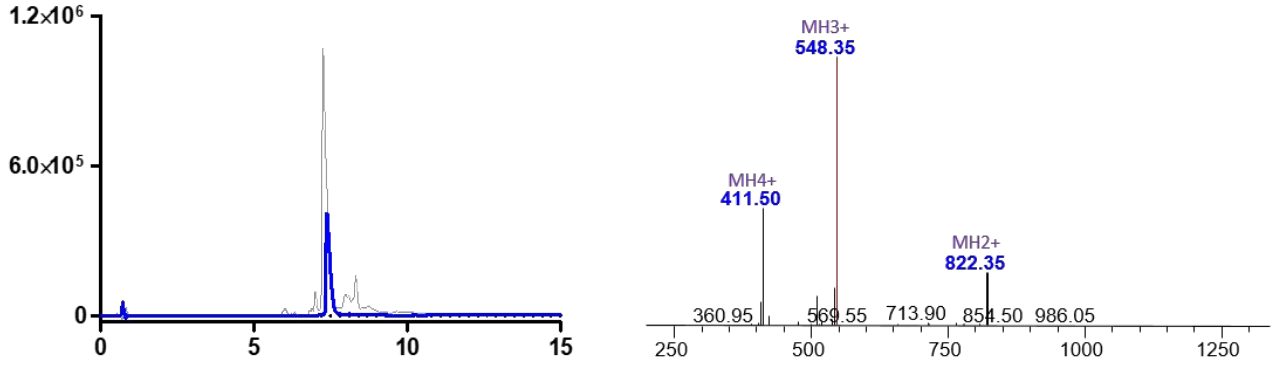

11: RLRWRKTLVLILVV

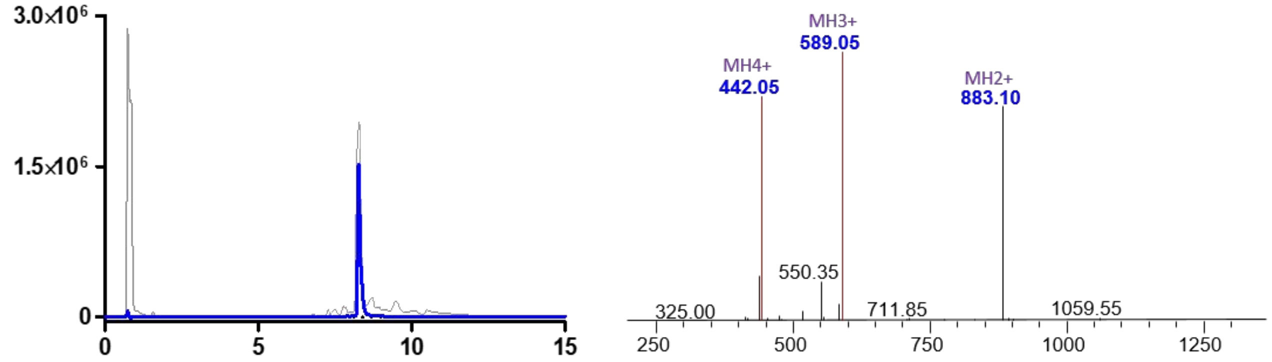

12: RKRKTLVLILVV

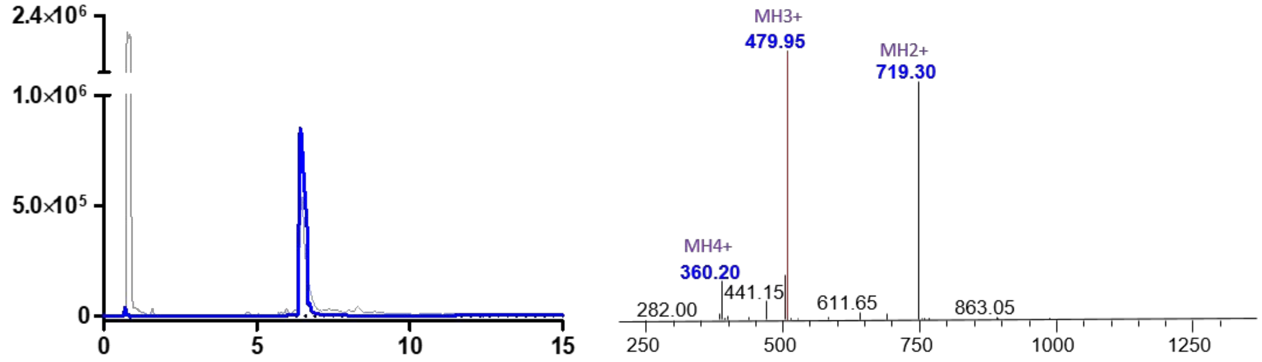

13: VYAYMYILWaGilkrw

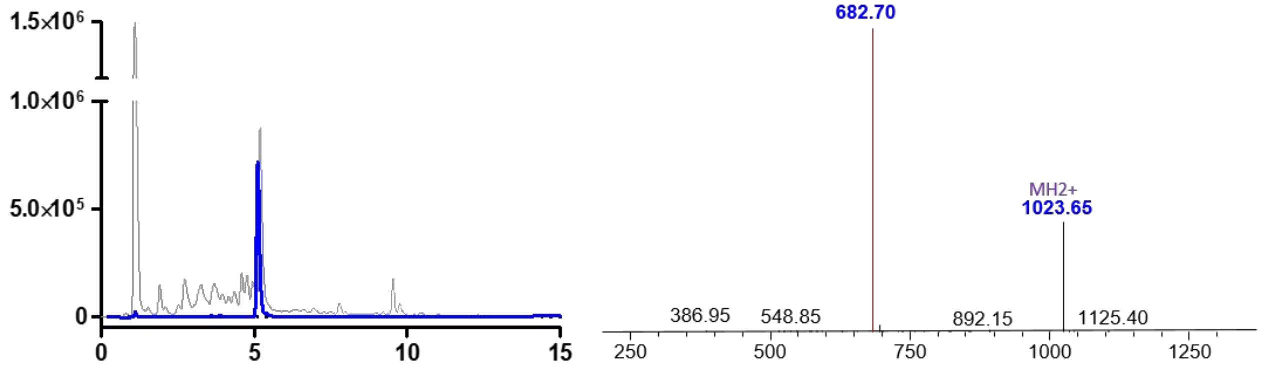

14: VYAYMYILWsksksk

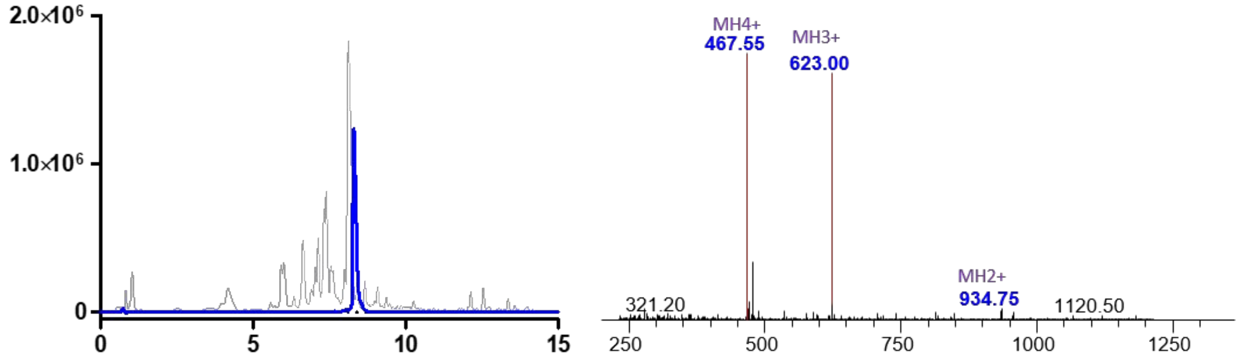

15: aGilkrwKTLVLILVV

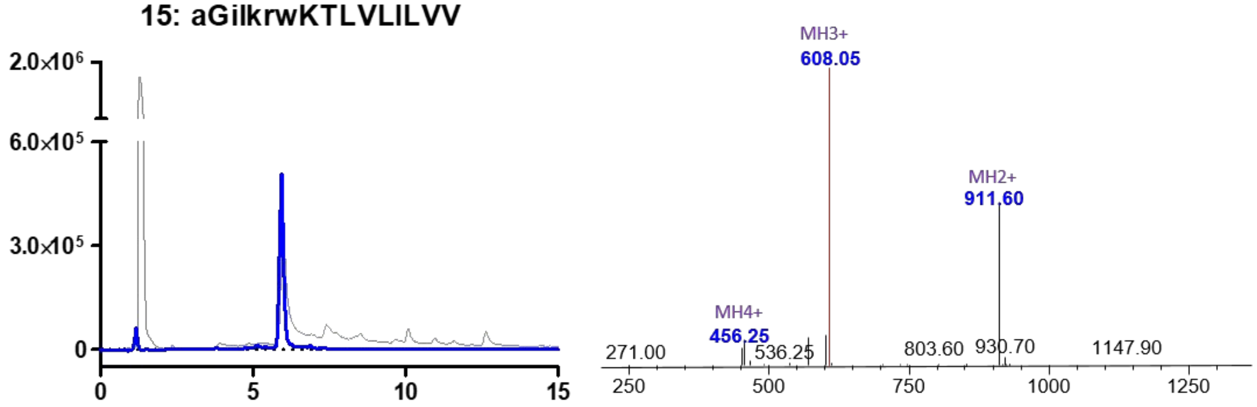

16: skskskKTLVLILVV

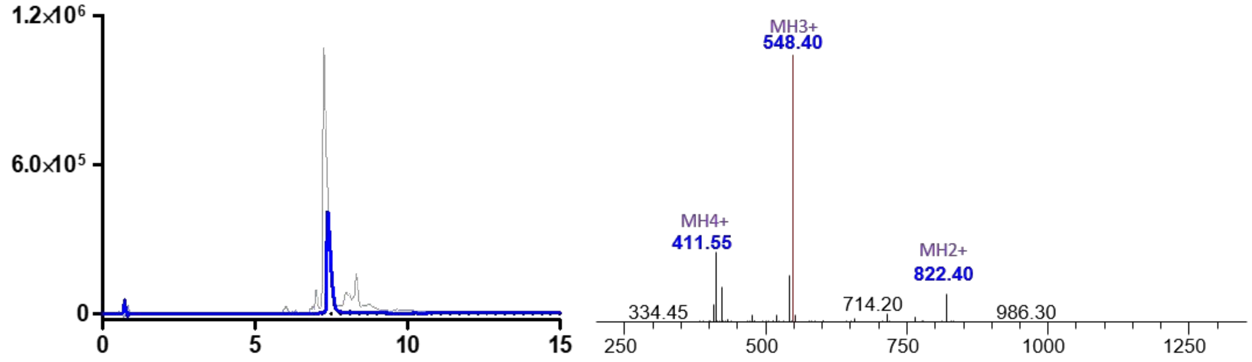

17: wliymyayvaGilkrw

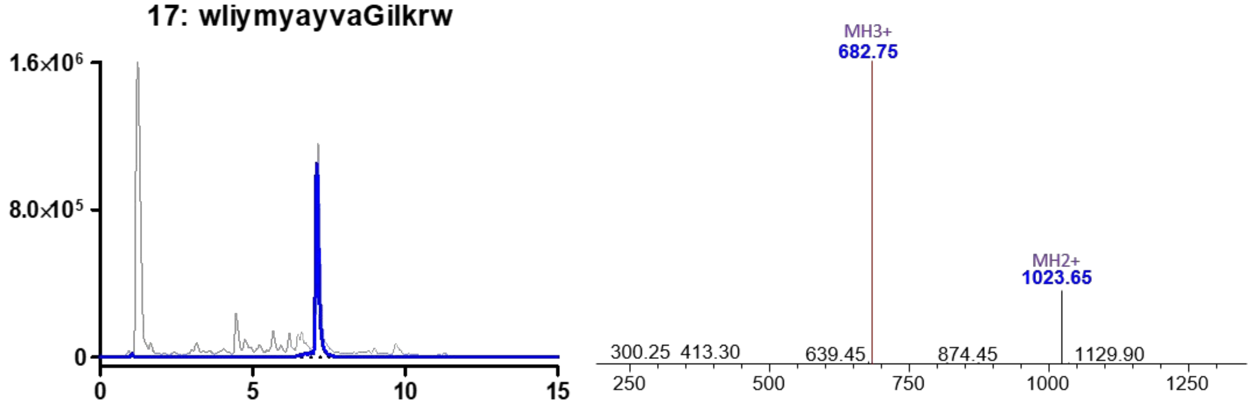

18: aGilkrwvvlilvltk

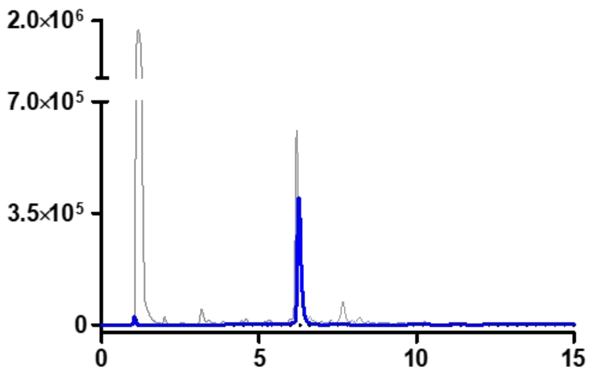

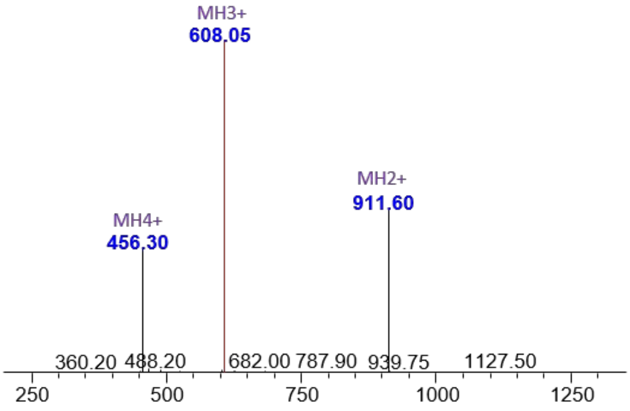

4. Molecular Formula Strings

SMILES notations. Molecular formula strings of Tables 1-4 compounds.

COMPOUND	SMILES NOTATIONS	MOLECULAR FORMULA
**TM5-Tat**	N[C@@H](CCC(O)=O)C(N[C@@H](C(C)O)C(N[C@@H](CC1=CC=C(O)C=C1)C(N[C@@H](CC(C)C)C(N[C@@H](CCSC)C(N[C@@H](CC2=CC=CC=C2)C(N[C@@H](CC3=CNC4=C3C=CC=C4)C(N[C@@H](C(CC)C)C(NC([H])C(N[C@@H](C(C)C)C(N[C@@H](C(C)O)C(N[C@@H](CO)C(N[C@@H]([C@H](C)C)C(N[C@@H](CC(C)C)C(N[C@@H](CC(C)C)C(N[C@@H](CC(C)C)C(N[C@@H](CC5=CC=CC=C5)C(N[C@@H](C(CC)C)C(N[C@@H](C(C)C)C(N[C@@H](CC6=CC=C(O)C=C6)C(N[C@@H](C)C(N[C@@H](CC7=CC=C(O)C=C7)C(N[C@@H](CCSC)C(N[C@@H](CC8=CC=C(O)C=C8)C(N[C@@H](C(CC)C)C(N[C@@H](CC(C)C)C(N[C@@H](CC9=CNC%10=C9C=CC=C%10)C(NC([H])C(N[C@@H](CCCNC(N)=N)C(N[C@@H](CCCCN)C(N[C@@H](CCCCN)C(N[C@@H](CCCNC(N)=N)C(N[C@@H](CCCNC(N)=N)C(N[C@@H](CCC(N)=O)C(N[C@@H](CCCNC(N)=N)C(N[C@@H](CCCNC(N)=N)C(N[C@@H](CCCNC(N)=N)C(N)=O)=O)=O)=O)=O)=O)=O)=O)=O)=O)=O)=O)=O)=O)=O)=O)=O)=O)=O)=O)=O)=O)=O)=O)=O)=O)=O)=O)=O)=O)=O)=O)=O)=O)=O)=O)=O	$C_{225}H_{351}N_{61}O_{47}S_2$
**Tat-TM6**	NC([H])C(N[C@@H](CCCNC(N)=N)C(N[C@@H](CCCCN)C(N[C@@H](CCCCN)C(N[C@@H](CCCNC(N)=N)C(N[C@@H](CCCNC(N)=N)C(N[C@@H](CCC(N)=O)C(N[C@@H](CCCNC(N)=N)C(N[C@@H](CCCNC(N)=N)C(N[C@@H](CCCNC(N)=N)C(N[C@@H](CCCCN)C(N[C@@H](C(C)O)C(N[C@@H](CC(C)C)C(N[C@@H]([C@@H](C)C)C(N[C@@H](CC(C)C)C(N[C@@H](C(CC)C)C(N[C@@H](CC(C)C)C(N[C@@H]([C@H](C)C)C(N[C@@H](C(C)C)C(N[C@@H](CC(C)C)C(N[C@@H](C(CC)C)C(N[C@@H](C(CC)C)C(N[C@H](CS)C(N[C@@H](CC1=CNC2=C1C=CC=C2)C(NC([H])C(N3[C@H](C(N[C@@H](CC(C)C)C(N[C@@H](CC(C)C)C(N[C@@H](C)C(N[C@@H](C(CC)C)C(N[C@@H](CCSC)C(N[C@@H](C(C)C)C(N[C@@H](CC4=CC=C(O)C=C4)C(N[C@@H](CC(O)=O)C(N[C@@H](C(C)C)C(N[C@@H](CC5=CC=CC=C5)C(O)=O)=O)=O)=O)=O)=O)=O)=O)=O)=O)=O)CCC3)=O)=O)=O)=O)=O)=O)=O)=O)=O)=O)=O)=O)=O)=O)=O)=O)=O)=O)=O)=O)=O)=O)=O)=O)=O	$C_{201}H_{345}N_{59}O_{42}S_2$

**TM7-Tat**	N[C@@H](CC(C)C)C(N[C@@H](C(CC)C)C(N[C@@H](CCCCN)C(N[C@@H](C(C)O)C(N[C@@H]([C@H](C)C)C(N[C@H](CC1=CC=CC=C1)C(N[C@@H](C)C(N[C@@H](CC2=CC=CC=C2)C(N[C@@H](CS)C(N[C@@H](CO)C(N[C@@H](CCSC)C(N[C@@H](CC(C)C)C(N[C@H](CS)C(N[C@H](CC(C)C)C(N[C@H](CC(C)C)C(N[C@H](CC(N)=O)C(N[C@H](CO)C(N[C@@H](C(C)O)C(N[C@H](C(C)C)C(N[C@H](CC(N)=O)C(N3[C@H]([C@@](N[C@@H](C(CC)C)C(N[C@@H](C(CC)C)C(N[C@H](CC4=CC=C(O)C=C4)C(N[C@H](C)C(N[C@H](CC(C)C)C(N[C@H](CCCNC(N)=N)C(NC([H])C(N[C@H](CCCNC(N)=N)C(N[C@H](CCCCN)C(N[C@H](CCCCN)C(N[C@H](CCCNC(N)=N)C(N[C@H](CCCNC(N)=N)C(N[C@H](CCC(N)=O)C(N[C@H](CCCNC(N)=N)C(N[C@H](CCCNC(N)=N)C(N[C@H](CCCNC(N)=N)C(N)=O)=O)=O)=O)=O)=O)=O)=O)=O)=O)=O)=O)=O)=O)=O)=O)=O)=O)CCC3)=O)=O)=O)=O)=O)=O)=O)=O)=O)=O)=O)=O)=O)=O)=O)=O)=O)=O)=O)=O)=O)=O	$C_{196}H_{339}N_{65}O_{45}S_3$
**1**	N[C@@H](C(C)C)C(N[C@@H](CC1=CC=C(O)C=C1)C(N[C@@H](C)C(N[C@@H](CC2=CC=C(O)C=C2)C(N[C@@H](CCSC)C(N[C@@H](CC3=CC=C(O)C=C3)C(N[C@@H](C(CC)C)C(N[C@@H](CC(C)C)C(N[C@@H](CC4=CNC5=C4C=CC=C5)C(NC([H])C(N[C@@H](CCCNC(N)=N)C(N[C@@H](CCCCN)C(N[C@@H](CCCCN)C(N[C@@H](CCCNC(N)=N)C(N[C@@H](CCCNC(N)=N)C(N[C@@H](CCC(N)=O)C(N[C@@H](CCCNC(N)=N)C(N[C@@H](CCCNC(N)=N)C(N[C@@H](CCCNC(N)=N)C(N)=O)=O)=O)=O)=O)=O)=O)=O)=O)=O)=O)=O)=O)=O)=O)=O)=O)=O)=O	$C_{118}H_{192}N_{42}O_{23}S$
**2**	N[C@@H]([C@@H](C)C)C(N[C@@H](CC1=CC=C(O)C=C1)C(N[C@@H](C)C(N[C@@H](CC2=CC=C(O)C=C2)C(N[C@@H](CCSC)C(N[C@@H](CC3=CC=C(O)C=C3)C(N[C@@H](C(CC)C)C(N[C@@H](CC(C)C)C(N[C@@H](CC4=CNC5=C4C=CC=C5)C(N)=O)=O)=O)=O)=O)=O)=O)=O)=O	$C_{63}H_{85}N_{11}O_{12}S$
**3**	NC([H])C(N[C@@H](CCCNC(N)=N)C(N[C@@H](CCCCN)C(N[C@@H](CCCCN)C(N[C@@H](CCCNC(N)=N)C(N[C@@H](CCCNC(N)=N)C(N[C@@H](CCC(N)=O)C(N[C@@H](CCCNC(N)=N)C(N[C@@H](CCCNC(N)=N)C(N[C@@H](CCCNC(N)=N)C(N[C@@H](CCCCN)C(N[C@@H](C(C)O)C(N[C@@H](CC(C)C)C(N[C@@H]([C@@H](C)C)C(N[C@@H](CC(C)C)C(N[C@@H](C(CC)C)C(N[C@@H](CC(C)C)C(N[C@@H]([C@@H](C)C)C(N[C@@H](C(C)C)C(N)=O)=O)=O)=O)=O)=O)=O)=O)=O)=O)=O)=O)=O)=O)=O)=O)=O)=O)=O	$C_{104}H_{200}N_{42}O_{21}$
**4**	O=C(N[C@@H](CC(C)C)C(N[C@@H](C(C)C)C(N[C@@H](CC(C)C)C(N[C@@H](C(CC)C)C(N[C@@H](CC(C)C)C(N[C@@H]([C@@H](C)C)C(N[C@@H](C(C)C)C(N)=O)=O)=O)=O)=O)=O)=O)[C@@H](NC([C@H](CCCCN)N)=O)C(C)O	$C_{49}H_{93}N_{11}O_{10}$

**5**	N[C@@H](C(C)C)C(N[C@@H](CC1=CC=C(O)C=C1)C(N[C@@H](C)C(N[C@@H](CC2=CC=C(O)C=C2)C(N[C@@H](CCSC)C(N[C@@H](CC3=CC=C(O)C=C3)C(N[C@@H](C(CC)C)C(N[C@@H](CC(C)C)C(N[C@@H](CC4=CNC5=C4C=CC=C5)C(N[C@@H](C)C(NC([H])C(N[C@@H](C(CC)C)C(N[C@@H](CC(C)C)C(N[C@@H](CCCCN)C(N[C@@H](CCCNC(N)=N)C(N[C@@H](CC6=CNC7=C6C=CC=C7)C(N)=O)=O)=O)=O)=O)=O)=O)=O)=O)=O)=O)=O)=O)=O)=O)=O	$C_{103}H_{149}N_{23}O_{19}S$
**6**	N[C@@H](C(C)C)C(N[C@@H](CC1=CC=C(O)C=C1)C(N[C@@H](C)C(N[C@@H](CC2=CC=C(O)C=C2)C(N[C@@H](CCSC)C(N[C@@H](CC3=CC=C(O)C=C3)C(N[C@@H](C(CC)C)C(N[C@@H](CC(C)C)C(N[C@@H](CC4=CNC5=C4C=CC=C5)C(N[C@@H](CO)C(N[C@@H](CCCCN)C(N[C@@H](CO)C(N[C@@H](CCCCN)C(N[C@@H](CO)C(N[C@@H](CCCCN)C(N)=O)=O)=O)=O)=O)=O)=O)=O)=O)=O)=O)=O)=O)=O	$C_{90}H_{136}N_{20}O_{21}S$
**7**	N[C@@H]([C@@H](C)C)C(N[C@@H](CC1=CC=C(O)C=C1)C(N[C@@H](C)C(N[C@@H](CC2=CC=C(O)C=C2)C(N[C@@H](CCSC)C(N[C@@H](CC3=CC=C(O)C=C3)C(N[C@@H](C(CC)C)C(N[C@@H](CC(C)C)C(N[C@@H](CC4=CNC5=C4C=CC=C5)C(N[C@@H](CCCNC(N)=N)C(N[C@@H](CC(C)C)C(N[C@@H](CCCNC(N)=N)C(N[C@@H](CC6=CNC7=C6C=CC=C7)C(N[C@@H](CCCNC(N)=N)C(N)=O)=O)=O)=O)=O)=O)=O)=O)=O)=O)=O)=O)=O	$C_{98}H_{142}N_{26}O_{17}S$
**8**	N[C@@H](C(C)C)C(N[C@@H](CC1=CC=C(O)C=C1)C(N[C@@H](C)C(N[C@@H](CC2=CC=C(O)C=C2)C(N[C@@H](CCSC)C(N[C@@H](CC3=CC=C(O)C=C3)C(N[C@@H](C(CC)C)C(N[C@@H](CC(C)C)C(N[C@@H](CC4=CNC5=C4C=CC=C5)C(N[C@@H](CCCNC(N)=N)C(N[C@@H](CCCCN)C(N[C@@H](CCCNC(N)=N)C(N)=O)=O)=O)=O)=O)=O)=O)=O)=O)=O)=O)=O	$C_{81}H_{121}N_{21}O_{15}S$
**9**	N[C@@H](C)C(NC([H])C(N[C@@H](C(CC)C)C(N[C@@H](CC(C)C)C(N[C@@H](CCCCN)C(N[C@@H](CCCNC(N)=N)C(N[C@@H](CC1=CNC2=C1C=CC=C2)C(N[C@@H](CCCCN)C(N[C@@H](C(C)O)C(N[C@@H](CC(C)C)C(N[C@@H](C(C)C)C(N[C@@H](CC(C)C)C(N[C@@H](C(CC)C)C(N[C@@H](CC(C)C)C(N[C@@H]([C@@H](C)C)C(N[C@@H]([C@@H](C)C)C(N)=O)=O)=O)=O)=O)=O)=O)=O)=O)=O)=O)=O)=O)=O)=O	$C_{89}H_{157}N_{23}O_{17}$

**10**	N[C@@H](CO)C(N[C@@H](CCCCN)C(N[C@@H](CO)C(N[C@@H](CCCCN)C(N[C@@H](CO)C(N[C@@H](CCCCN)C(N[C@@H](CCCCN)C(N[C@@H](C(C)O)C(N[C@@H](CC(C)C)C(N[C@@H]([C@@H](C)C)C(N[C@@H](CC(C)C)C(N[C@@H](C(CC)C)C(N[C@@H](CC(C)C)C(N[C@@H]([C@@H](C)C)C(N[C@@H](C(C)C)C(N)=O)=O)=O)=O)=O)=O)=O)=O)=O)=O)=O)=O)=O)=O)=O	$C_{76}H_{144}N_{20}O_{19}$
**11**	N[C@@H](CCCNC(N)=N)C(N[C@@H](CC(C)C)C(N[C@@H](CCCNC(N)=N)C(N[C@@H](CC1=CNC2=C1C=CC=C2)C(N[C@@H](CCCNC(N)=N)C(N[C@@H](CCCCN)C(N[C@@H](C(C)O)C(N[C@@H](CC(C)C)C(N[C@@H](C(C)C)C(N[C@@H](CC(C)C)C(N[C@@H](C(CC)C)C(N[C@@H](CC(C)C)C(N[C@@H](C(C)C)C(N[C@@H]([C@H](C)C)C(N)=O)=O)=O)=O)=O)=O)=O)=O)=O)=O)=O)=O)=O	$C_{84}H_{150}N_{26}O_{15}$
**12**	N[C@@H](CCCNC(N)=N)C(N[C@@H](CCCCN)C(N[C@@H](CCCNC(N)=N)C(N[C@@H](CCCCN)C(N[C@@H](C(C)O)C(N[C@@H](CC(C)C)C(N[C@@H]([C@@H](C)C)C(N[C@@H](CC(C)C)C(N[C@@H](C(CC)C)C(N[C@@H](CC(C)C)C(N[C@@H]([C@@H](C)C)C(N[C@@H](C(C)C)C(N)=O)=O)=O)=O)=O)=O)=O)=O)=O)=O)=O)=O	$C_{67}H_{129}N_{21}O_{13}$
**13**	N[C@@H](C(C)C)C(N[C@@H](CC1=CC=C(O)C=C1)C(N[C@@H](C)C(N[C@@H](CC2=CC=C(O)C=C2)C(N[C@@H](CCSC)C(N[C@@H](CC3=CC=C(O)C=C3)C(N[C@@H](C(CC)C)C(N[C@@H](CC(C)C)C(N[C@@H](CC4=CNC5=C4C=CC=C5)C(N[C@H](C)C(NC([H])C(N[C@H](C(CC)C)C(N[C@H](CC(C)C)C(N[C@H](CCCCN)C(N[C@H](CCCNC(N)=N)C(N[C@H](CC6=CNC7=C6C=CC=C7)C(N)=O)=O)=O)=O)=O)=O)=O)=O)=O)=O)=O)=O)=O)=O)=O)=O	$C_{103}H_{149}N_{23}O_{19}S$
**14**	N[C@@H](C(C)C)C(N[C@@H](CC1=CC=C(O)C=C1)C(N[C@@H](C)C(N[C@@H](CC2=CC=C(O)C=C2)C(N[C@@H](CCSC)C(N[C@@H](CC3=CC=C(O)C=C3)C(N[C@@H](C(CC)C)C(N[C@@H](CC(C)C)C(N[C@@H](CC4=CNC5=C4C=CC=C5)C(N[C@H](CO)C(N[C@H](CCCCN)C(N[C@H](CO)C(N[C@H](CCCCN)C(N[C@H](CO)C(N[C@H](CCCCN)C(N)=O)=O)=O)=O)=O)=O)=O)=O)=O)=O)=O)=O)=O)=O)=O	$C_{90}H_{136}N_{20}O_{21}S$

**15**	N[C@H](C)C(NC([H])C(N[C@H](C(CC)C)C(N[C@H](CC(C)C)C(N[C@H](CCCCN)C(N[C@H](CCCNC(N)=N)C(N[C@H](CC1=CNC2=C1C=CC=C2)C(N[C@@H](CCCCN)C(N[C@@H](C(C)O)C(N[C@@H](CC(C)C)C(N[C@@H](C(C)C)C(N[C@@H](CC(C)C)C(N[C@@H](C(CC)C)C(N[C@@H](CC(C)C)C(N[C@@H]([C@@H](C)C)C(N[C@@H]([C@@H](C)C)C(N)=O)=O)=O)=O)=O)=O)=O)=O)=O)=O)=O)=O)=O)=O)=O	$C_{89}H_{157}N_{23}O_{17}$
**16**	N[C@H](CO)C(N[C@H](CCCCN)C(N[C@H](CO)C(N[C@H](CCCCN)C(N[C@H](CO)C(N[C@H](CCCCN)C(N[C@@H](CCCCN)C(N[C@@H](C(C)O)C(N[C@@H](CC(C)C)C(N[C@@H](C(C)C)C(N[C@@H](CC(C)C)C(N[C@@H](C(CC)C)C(N[C@@H](CC(C)C)C(N[C@@H]([C@@H](C)C)C(N[C@@H](C(C)C)C(N)=O)=O)=O)=O)=O)=O)=O)=O)=O)=O)=O)=O)=O)=O	$C_{76}H_{144}N_{20}O_{19}$
**17**	N[C@H](CC1=CNC2=C1C=CC=C2)C(N[C@H](CC(C)C)C(N[C@H](C(CC)C)C(N[C@H](CC3=CC=C(O)C=C3)C(N[C@H](CCSC)C(N[C@H](CC4=CC=C(O)C=C4)C(N[C@H](C)C(N[C@H](CC5=CC=C(O)C=C5)C(N[C@H]([C@@H](C)C)C(N[C@H](C)C(NC([H])C(N[C@H](C(CC)C)C(N[C@H](CC(C)C)C(N[C@H](CCCCN)C(N[C@H](CCCNC(N)=N)C(N[C@H](CC6=CNC7=C6C=CC=C7)C(N)=O)=O)=O)=O)=O)=O)=O)=O)=O)=O)=O)=O)=O)=O)=O	$C_{103}H_{149}N_{23}O_{19}S$
**18**	N[C@H](C)C(NC([H])C(N[C@H](C(CC)C)C(N[C@H](CC(C)C)C(N[C@H](CCCCN)C(N[C@H](CCCNC(N)=N)C(N[C@H](CC1=CNC2=C1C=CC=C2)C(N[C@H](C(C)C)C(N[C@H](C(C)C)C(N[C@H](CC(C)C)C(N[C@H](C(CC)C)C(N[C@H](CC(C)C)C(N[C@H](C(C)C)C(N[C@H](CC(C)C)C(N[C@H](C(C)O)C(N[C@H](CCCCN)C(N)=O)=O)=O)=O)=O)=O)=O)=O)=O)=O)=O)=O)=O)=O)=O	$C_{89}H_{157}N_{23}O_{17}$
